# Integrative Epigenomic Analysis of Transcriptional Regulation of Human CircRNAs

**DOI:** 10.3389/fgene.2020.590672

**Published:** 2021-01-25

**Authors:** Xue-Cang Li, Zhi-Dong Tang, Li Peng, Yan-Yu Li, Feng-Cui Qian, Jian-Mei Zhao, Ling-Wen Ding, Xiao-Juan Du, Meng Li, Jian Zhang, Xue-Feng Bai, Jiang Zhu, Chen-Chen Feng, Qiu-Yu Wang, Jian Pan, Chun-Quan Li

**Affiliations:** ^1^School of Medical Informatics, Harbin Medical University, Daqing, China; ^2^Guangdong Province Key Laboratory of Malignant Tumor Epigenetics and Gene Regulation, Sun Yat-sen Memorial Hospital, Sun Yat-sen University, Guangzhou, China; ^3^Cancer Science Institute of Singapore, National University of Singapore, Singapore, Singapore; ^4^The 942 Hospital of Joint Logistic Support Force of PLA, Yinchuan, China; ^5^Department of Hematology and Oncology, Children's Hospital of Soochow University, Suzhou, China

**Keywords:** transcription factors, transcriptional regulation, epigenomic, circular RNAs, TAH-circRNAs

## Abstract

Circular RNAs (circRNAs) are evolutionarily conserved and abundant non-coding RNAs whose functions and regulatory mechanisms remain largely unknown. Here, we identify and characterize an epigenomically distinct group of circRNAs (TAH-circRNAs), which are transcribed to a higher level than their host genes. By integrative analysis of cistromic and transcriptomic data, we find that compared with other circRNAs, TAH-circRNAs are expressed more abundantly and have more transcription factors (TFs) binding sites and lower DNA methylation levels. Concordantly, TAH-circRNAs are enriched in open and active chromatin regions. Importantly, ChIA-PET results showed that 23–52% of transcription start sites (TSSs) of TAH-circRNAs have direct interactions with cis-regulatory regions, strongly suggesting their independent transcriptional regulation from host genes. In addition, we characterize molecular features of super-enhancer-driven circRNAs in cancer biology. Together, this study comprehensively analyzes epigenomic characteristics of circRNAs and identifies a distinct group of TAH-circRNAs that are independently transcribed via enhancers and super-enhancers by TFs. These findings substantially advance our understanding of the regulatory mechanism of circRNAs and may have important implications for future investigations of this class of non-coding RNAs.

## Introduction

CircRNAs are a large class of non-coding RNAs produced by circularization of exons, and they are characterized by the presence of a covalent direct ligation of 3′ and 5′ ends generated by back splicing (Chen, 2016). Although the vast majority of circRNAs have not been studied, several lines of evidence have suggested that this group of non-coding RNAs play important roles in tissue development (You et al., 2015), gene regulation, and carcinogenesis (Hsiao et al., 2017). With the advent of high-throughput profiling technologies, a myriad of circRNAs have recently been identified across various types of cells, tissues, and species (Zhang X. O. et al., 2016; Dong et al., 2017). Intriguingly, although the expression levels of the majority of circRNAs are expectedly low, some of them are transcribed at comparable or even higher levels than their linear counterparts (Li X. et al., 2017). For example, circRNAs are highly abundant in the fly brain (Westholm et al., 2014), mammalian neuronal and muscle tissues (Rybak-Wolf et al., 2015).

Although it is generally assumed that circRNAs are the spliced products of their host transcripts, a few lines of evidence suggested that some circRNAs may be transcriptionally activated independently from their host genes. For example, a previous study found that expression changes of a group of circRNAs were largely independent of host genes during the differentiation of epidermal stem cells (Kristensen et al., 2018). Moreover, the transcription factor Twist1 selectively promoted the transcription of circRNA Cul2 but not its host gene (Meng et al., 2018). In addition, Bin et al. observed that circRNA Nfix was driven by a super-enhancer (SE) in cardiomyocyte cells, which was also independent from its host gene (Huang et al., 2019). Interestingly, during neuronal differentiation, some circRNAs were up-regulated to a higher extent compared to their host mRNAs (You et al., 2015). However, it is unclear whether these observations represent merely particular cases or there exists a general regulatory mechanism for independent transcription of circRNAs.

To address this question, we performed comprehensive epigenomic analyses of circRNAs and identified a distinct group of these non-coding RNAs that are transcriptionally activated to a higher level than the host genes (TAH-circRNAs). Specific TFs occupy genomic regions of these TAH-circRNAs, which exhibit higher conservation than other circRNAs. Cis-regulatory regions of TAH-circRNAs have increased active histone modifications and reduced DNA methylation. Furthermore, TAH-circRNAs associated with SE have higher expression level and denser TF occupancy than other circRNAs. Lastly, we functionally validated a group of TAH-circRNAs that were independently transcribed by a master regulator TF, FOXA1. These results together provide many new insights into transcription regulatory mechanism of circRNAs.

## Materials and Methods

### Cell Culture

HepG2 cells were maintained in Dulbecco's modified Eagle's medium (DMEM) supplemented with 10% fetal bovine serum (FBS). Cells were incubated at 37°C in a 5% CO_2_.

### siRNA Transfection

Three different siRNAs against human FOXA1 (Supplementary Table 3) were designed and ordered from GenePharma (Shanghai, China). Synthetic sequence-scrambled siRNA was used as a negative control (NC). HepG2 cells were seeded in six-well plate until cell density reached 30–40% prior to siRNA transfection. Three siRNAs were mixed and transfected using Lipofectamine RNAiMAX (Invitrogen, USA) and incubated for 48 h.

### Real-Time PCR Detection

Total RNA was isolated from HepG2 cells using TRIzol (Invitrogen, USA) and was reverse-transcribed to cDNA with RevertAid RT Reverse Transcription Kit (Thermo Scientific, USA). Real-time PCR was performed with SYBR Green I Master (Roche, USA) in LightCycler 480 System. The sequences of the PCR primers were listed in Supplementary Table 3. All samples were measured in triplicate and normalized by GAPDH. The data were expressed as the mean ± SD of three independent experiments.

To validate the sequences of circRNAs in HepG2 cells, the products of real-time PCR were sequenced by Sanger method and the results completely matched with the predicted sequences.

### Microarray

Agilent Human V6 (4^*^180K, Design ID: 084410) was used for hybridization of total RNAs and was then scanned by Agilent Scanner G2505C (Agilent Technologies, USA) to measure mRNA and circRNA.

### Luciferase Reporter Assay

Hsa_circ_0061137_upstream + 1,500 nt and hsa_circ_0061137_upstream + 1,000 nt were cloned into psicheck2 luciferase reporter vector (Promega). Luciferase vector and control vector were transfected into HepG2 cells with Lipofectamine 3000 (Thermo Fisher Scientific). After 48 h of transfection, the reporter activity was measured by the Dual-Luciferase Reporter Assay System (Promega, E1910).

### Genomic Annotation of CircRNAs and Coding Genes

Genomic annotations of 92,375 human circRNAs and 20,287 coding genes were downloaded from circBase database (December 15, 2015, http://www.circbase.org/) and ensemble (GRCh37, http://grch37.ensembl.org/index.html), respectively.

### Identification of CircRNAs and Quantification of the Expression Levels of CircRNAs and Coding Genes

We downloaded ENCODE RNA-seq data of 36 cell lines, including PolyA- RNA-seq data of 17 cell lines and Total RNA-seq data of 19 cell lines (Supplementary Table 1). The find_circ (Memczak et al., 2013) computational pipeline was applied to identify circRNAs for each cell line. These identified circRNAs were next annotated to circBase database. A circRNA was assigned to the corresponding circBase ID if it had the same positions with an annotated circRNA in the database. We computed the expression level of circRNAs by different methods: (1) CircRNAs expression was normalized as the number of back-splice junction spanning reads per million raw reads (RPM). Since longer reads will have more power in detecting back-splicing events, read length can be used as normalization for circRNAs. Because find_circ software requires anchor sequences of 20 bp on each side of the read, a reads per kilobase per million mapped reads (RPKM) expression value for each circRNA was calculated by dividing circRNAs RPM value by 112 and multiplying by 1,000 (Veno et al., 2015).

(2) We computed the expression of gene body of circRNAs using the following equation:

$$\begin{array}{l} \text{RPK}{\text{M}}_{body}\text{=}\frac{10^{9}*m}{n*L} \end{array}$$

Where *m* corresponds to the number of reads locating in gene body of circRNAs; *n* corresponds to the total number of reads; and *L* represents the length of circRNAs.

(3) H3K27ac modification is associated with the regulatory regions of active genes. Therefore, we considered that if the proximal regulatory region of a circRNA has higher H3K27ac signals than other circRNAs, it is likely to have higher transcriptional activity. To measure the transcriptional activity of circRNAs, we normalized H3K27ac signals within proximal regulatory region of circRNAs, which is defined as a region that spread 1,000 bp upstream and 1,000 bp downstream from its TSS. The transcriptional activity of a circRNA was measured as:

$$\begin{array}{l} {\text{RPKM}}_{ss}=\frac{10^{9}*C}{2000*N} \end{array}$$

Where *C* corresponds to the number of H3K27ac reads locating in regulatory region of the circRNA; *N* corresponds to the total number of reads.

### Genome-Wide Binding Analysis of TFs

A total of 690 TF ChIP-seq data were downloaded from ENCODE. The peak list of these ChIP-seq data was based on 161 unique TFs, generated from uniformly processed pipeline. This dataset spans 91 human cell types, where the calling method of SPP (Kharchenko et al., 2008) was used to identify the regions of enrichment (peaks) (Supplementary Table 1). In addition, we used ChIP-seq data of histone marks and DNaseI.

Moreover, we analyzed ChIP-seq data of H3K27ac histone mark from Hnisz et al. (2013), which included eight (GM12878, H1-hESC, HeLa-S3, HepG2, HUVEC, IMR90, K562, MCF-7) cell lines.

### DNA Methylation Analysis

We downloaded processed beta-values of DNA methylation 450 k array (61 cell lines) and whole-genome bisulfite sequencing (WGBS) data (four cell lines) from ENCODE. We converted the genome coordinate of WGBS (GRCh38) data to hg19 through liftover (https://genome.ucsc.edu/cgi-bin/hgLiftOver). For WGBS dataset, only CpGs with a minimal coverage of 4 were included for analysis.

### Construction of TF-Mediated Regulatory Network of CircRNAs and Coding Genes

For each of 92,375 circRNAs from circBase, we identified those TFs that occupy the regulatory regions of circRNAs through searching 161 TF ChIP-seq data from ENCODE. A circRNA is considered as occupied by a TF if its regulatory region (−2/+1 Kb of its TSS) has at least one peak. We merged all the identified TF-circRNA pairs to a global regulatory network of TF-circRNA. In parallel, we also constructed regulatory network of TF-coding genes by the method. According to the global regulatory network of TF-circRNA, we abstracted a regulatory sub network of TAH-circRNAs through mapping TAH-circRNA and abstracting nearest TFs of them.

### ChIP-Seq Data Processing and Identifying ChIP-Seq Enriched Region in Human Cells

Sequencing reads were aligned to human genome build hg19 using bowtie 0.12.9 using parameters -K 2 -m 2 -n 2 -S. Enrichment regions of H3K27ac and TF in human sample were calculated using MACS 1.4.2 (Zhang et al., 2008; Jiang et al., 2019; Qian et al., 2019; Tang et al., 2019; Feng et al., 2020; Li et al., 2020) using parameters --keep-dup 1 --wig --single-profile--space = 50 -p 1e-9 on H3K27ac and various TF ChIP-seq with control libraries. UCSC Genome Browser tracks were generated with MACS wiggle outputs. MACS peaks of human H3K27ac were used as constituent enhancers for SE identification. ChIP signal visualization was done by IGV (Integrative Genomics Viewer) (Thorvaldsdottir et al., 2013) and the input WIG files were created by MACS.

### Identification of SE and Associated CircRNAs

SE were identified using ROSE (https://bitbucket.org/young_computation/rose) with parameters -s 12,500 -t 2,000 by stitching enhancers, which has been described previously (Loven et al., 2013). Typical-enhancer and SE associated circRNAs were identified using a Python script of ROSE.

### CircRNA Microarray of HCC Samples

We re-annotated and re-analyzed the circRNA expression profiles of HCC sample from GEO GSE97332, which was based on Agilent circRNA microarray. Specifically, we created a library of circRNA junction sequences containing 45 bp from tail and 45 bp from head of each circRNA in circBase. We then performed re-annotation by aligning the probe sequences of the Agilent circRNA microarray to our customized junction library with BLASTn tools (McGinnis and Madden, 2004). Results from the sequence alignment were further processed as follows: (1) we retained the probes matching to only one unique circRNA, resulting in a set of probe-circRNA pairs. (2) Each circRNA is required to be perfectly matched to at least one probe. As a result, a total of 5,515 circBase circRNAs were re-annotated.

### ChIA-PET

We downloaded the RAD21, CTCF, POLR2A, and ESR1 ChIA-PET data in three cell lines (HepG2, K562, and MCF7) from ENCODE. We then analyzed these datasets by ChIA-PET2 (Li G. et al., 2017) using parameters -e 1 -k 2. In addition, we downloaded the chromatin interaction data of GM12787 and IMR90 from 4DGenome and extracted the chromatin interactions that were detected by 3C, 5C, and Hi-C.

### Gene Set Enrichment Analysis (GSEA)

GSEA was performed using the tool from http://software.broadinstitute.org/gsea/index.jsp. In brief, *t*-test in gene expression from two experimental conditions was calculated. The result was used as a ranked list in the Pre-Ranked function of the GSEA software. Expression matrixes of circRNAs and coding genes from siFOXA1 (FOXA1 knockdown) and control group were created. Gene sets based on the TAH-circRNAs and host genes were generated in the HepG2 cells.

### Permutation Test

Permutation test was performed by sampling 20,000 times random circRNA sets, each with the same size as the test circRNA set (1,201). For each random set, we counted the number of circRNAs that overlap with down-regulated circRNAs.

## Results

### Identification of a Group of TAH-CircRNAs With Distinct Epigenomic Features

**Figure 1:**
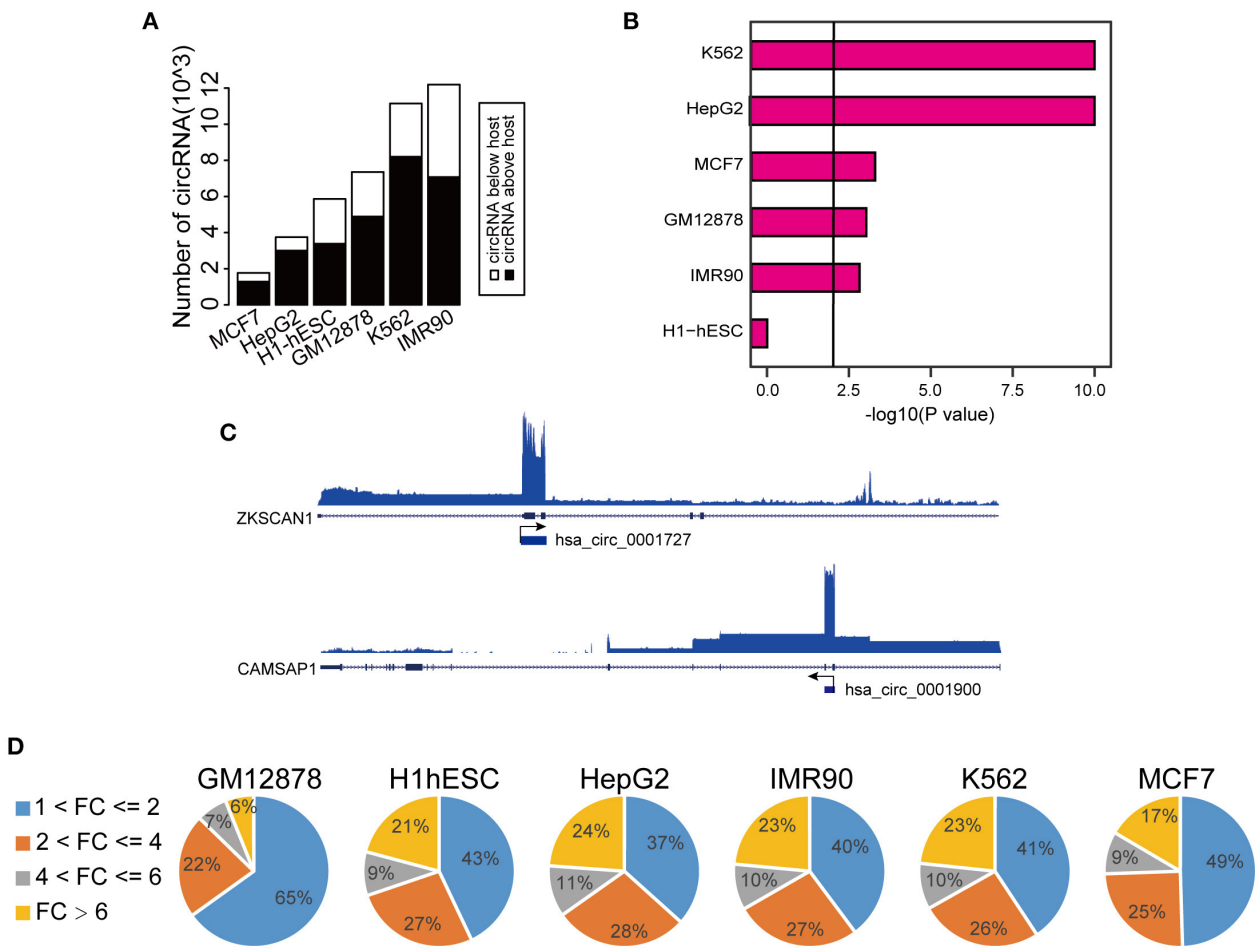
Expression analysis of circRNAs. **(A)** Global expression patterns of circRNAs compared to their host genes in different ENCODE cell lines. **(B)** Significance of permutation test by randomly generating 10,000 independent circRNA sets in each cell lines. **(C)** IGV (Integrative Genomics Viewer) plots of RNA-seq profiles has_circ_0001727 and has_circ_0001900 and their host genes. **(D)** Fold changes of circRNAs with higher expression level than their host genes in each cell lines.

To comprehensively determine the expression profiles of circRNAs, we first annotated all circRNAs in 36 cell lines (with matched Total RNA-seq and Poly-A RNA-seq data) using the Find_circ software, and computed the RPKM (Reads Per Kilobase Million) values of both circRNAs and the host genes. We then focused on six cell lines (GM12878, H1-hESC, K562, HepG2, IMR90, and MCF7) from ENCODE consortium since they have comprehensive epigenomic data, including profilings of TF occupancies, histone modifications, and DNA methylation. We found that a significant proportion of circRNAs (40–60% across different cell lines) had higher RPKM compared to their host genes (Figure 1A and Supplementary Figures 1A–G), in line with previous reports (Salzman et al., 2013; Rybak-Wolf et al., 2015; You et al., 2015; Zhang Y. et al., 2016; Legnini et al., 2017; Kristensen et al., 2018). Moreover, this increase over host genes was only observed in circRNAs specifically identified by Find_circ but not in predicted circRNAs using circBase (Figure 1B), suggesting the specificity of the expression difference between circRNAs and host genes. As examples shown in Figure 1C, the level of hsa_circ_0001727 and hsa_circ_0001900 was ~15 and ~43 times higher than their host genes, respectively. Indeed, as much as 6–24% of circRNAs had at least 6-fold higher expression levels than their hosts (Figure 1D). At the same time, we calculated the number of TAH-circRNA's exons and their host genes in six cell lines, respectively (Supplementary Figure 7). The distribution of TAH-circRNA's exons were different from their host genes. Most of TAH-circRNAs contained one or two exons, whereas their host genes were composed of multi-exons. For instance, hsa_circ_0001727 was originated from exon 2 and exon 3 of the host gene (contained six exons, ZKSCAN1), and the expression level of this TAH-circRNA was higher than its host gene, consistent with the result found by Yao et al. (2017). Hsa_circ_0001900 originated from exon 2 and exon 3 of the host gene (contained 17 exons, CAMSAP1). Zhu et al. measured circ_0001900 and found that the circRNA was significantly up-regulated in CRC tissues and much more stable than the host gene (CAMSAP1) (Zhou et al., 2020). Taken together, these results indicated that TAH-circRNAs exhibit the different transcription pattern of exons compared with host gene.

**Figure 2:**
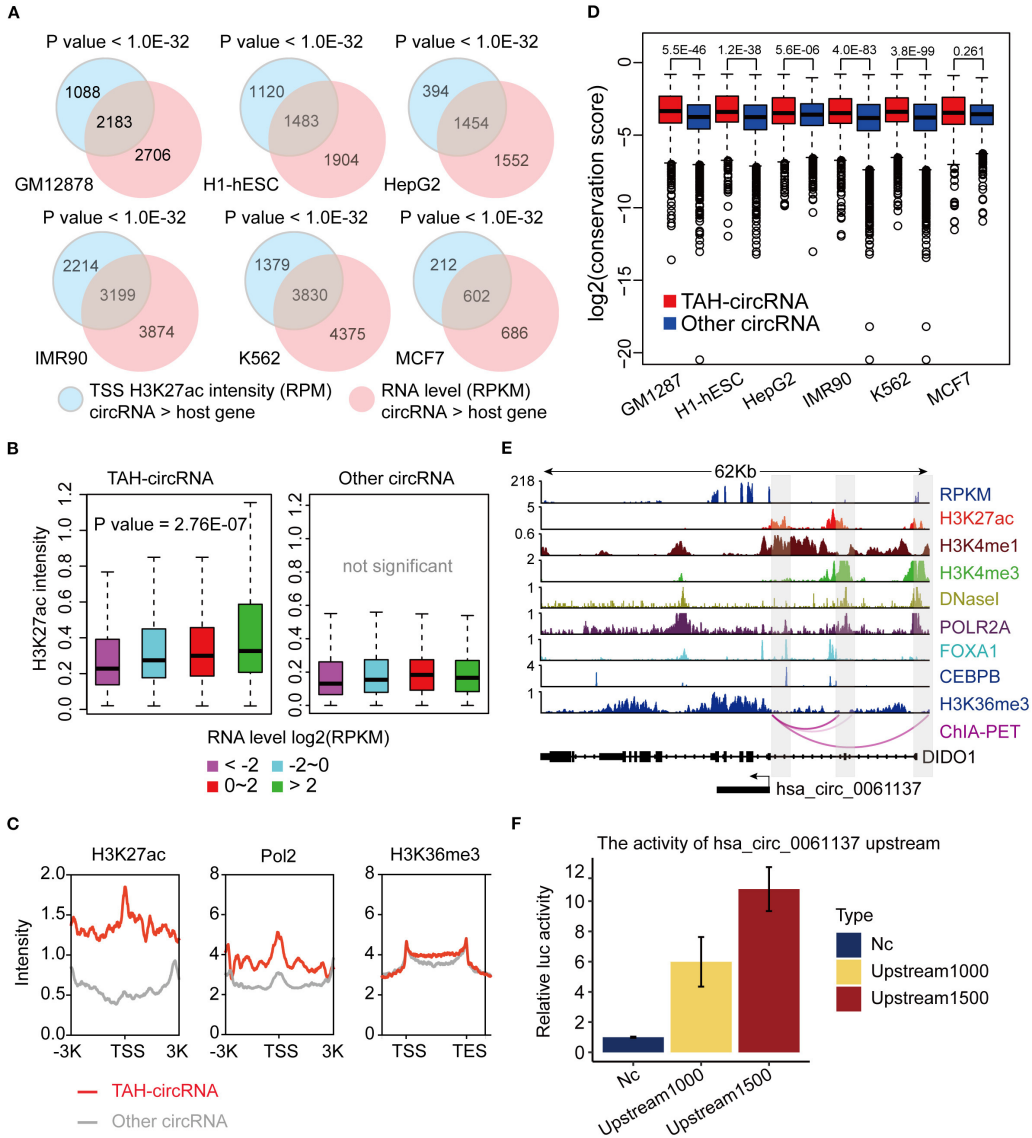
Epigenomic analyses of TAH-circRNAs in human cell lines. **(A)** Venn diagram showing the overlap between increased H3K27ac signal intensity in proximal regulatory region of circRNAs, *P*-value was performed using hypergeometric test. **(B)** Box plots showing the correlation between H3K27ac signal intensity in proximal regulatory region of circRNAs and RPKM level of circRNAs, *P*-value was calculated by Pearson correlation. **(C)** Metagene representation of indicated ChIP-seq occupancy at either TAH-circRNAs or other circRNAs in HepG2 cell line. TSS, transcription start site of circRNAs. **(D)** Conservation scores of circRNAs in indicated cell lines. **(E)** IGV plots of RNA-seq and ChIP-seq profiles for the indicated histone marks and TFs for hsa_circ_0061137 and its host gene DIDO1 in HepG2 cell line. **(F)** The transcriptional activity of TSS (hsa_circ_0061137_upstream 1,500/1,000 nt) was assessed by luciferase reporter assay in HepG2.

To explore the transcriptional activity of circRNAs with higher expression levels than the hosts, we next analyzed H3K27ac (a histone marker indicative of active transcription) ChIP-seq profiles from these six cell lines. Specifically, we measured H3K27ac intensity in potential proximal regulatory regions of circRNAs (between 2 Kb upstream and 1 Kb downstream of the circRNA TSS). Consistent with the changes in RNA expression, we observed that 30–40% circRNAs had higher H3K27ac signals than the host genes (Supplementary Figure 1H). Importantly, circRNAs with higher H3K27ac signals strongly overlapped with those having higher expression levels (Figure 2A, Supplementary Table 2). In fact, 17–32% of circRNAs with higher H3K27ac signals had higher expression levels, strongly suggesting that the increased level of abundance of these circRNAs is not simply due to back-splicing. We considered that circRNAs with both higher H3K27ac signals and elevated expression levels might potentially have unique transcriptional mechanism, and thus we focused on characterizing this group, which we termed TAH-circRNAs (Transcriptionally Activated to a Higher level than host genes). TAH-circRNAs accounted for 20–30% of all circRNAs across various cell lines (Supplementary Figure 2A). Importantly, a significantly positive correlation between the expression level and the H3K27ac intensity was observed only in TAH-circRNAs (Figure 2B), but not in non-TAH-circRNAs. Furthermore, TAH-circRNAs had both higher H3K36me3 signals and POLR2A loadings than other circRNAs in each cell line (Figure 2C, Supplementary Figures 2B,D–F). Consistently, TAH-circRNAs displayed more elevated DNaseI signals than non-TAH-circRNAs (Supplementary Figure 2C). Lastly, we observed that TAH-circRNAs were more evolutionarily conserved than other circRNAs (Figure 2D), implying that TAH-circRNAs might have more important functions and thus were more conserved across species. Figure 2E shows one example of TAH-circRNAs (hsa_circ_0061137), which had higher levels of H3K4me1, H3K36me3, as well RPKM values than the host gene (DIDO1). Most importantly, ChIA-PET results identified direct interactions between the TSS of this circRNA and two distal cis-regulatory regions. These two distal regions had conspicuous H3K27ac modification, DNase I signals, as well as POLR2A binding, indicative of enhancer function. Furthermore, FOXA1 and CEBPB were found to occupy both its TSS and one of the cis-regulatory regions, implying that this circRNA might be under direct transcriptional regulation from these two TFs. Other example TAH-circRNAs are shown in Supplementary Figures 6A–D. The existence of hsa_circ_0061137 promoter was further experimental verified by luciferase reporter assay in HepG2, which showed the high transcriptional activity of hsa_circ_0061137 transcriptional start site (upstream 1,500/1,000 nt) (Figure 2F). Taken together, these analyses distinguished TAH-circRNAs from other circRNAs in terms of epigenomic and biological features.

### Regulatory Regions of TAH-CircRNA Have Low DNA Methylation

**Figure 3:**
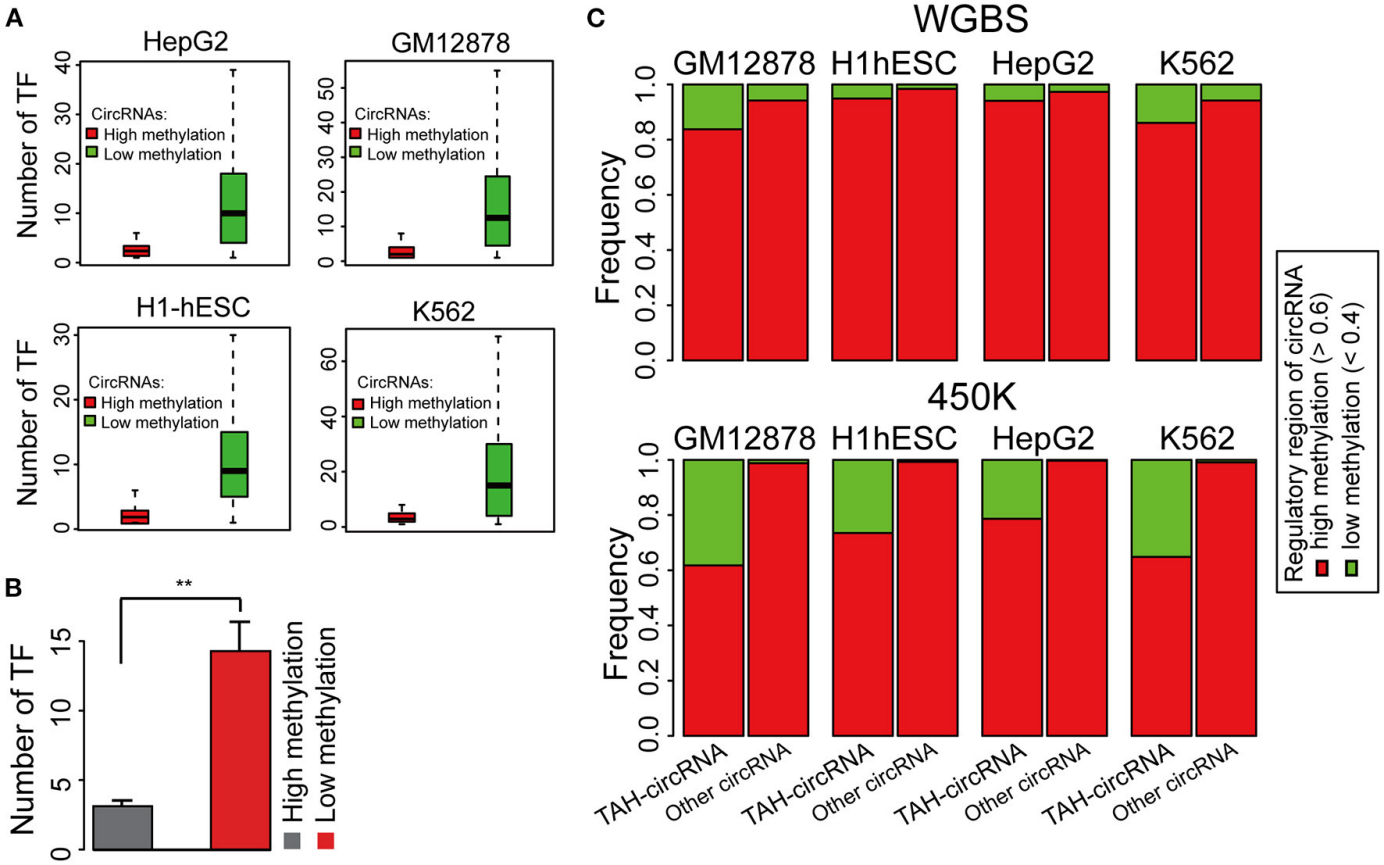
DNA Methylation pattern of the proximal regulatory regions circRNAs. **(A)** The number of TFs occupying circRNAs in indicated cell lines stratified by the DNA methylation level of the proximal regulatory region of circRNAs using WGBS data. **(B)** Average number of TFs that occupy circRNAs stratified by the DNA methylation level of the proximal regulatory region of circRNAs using WGBS data. **(C)** DNA methylation pattern of the proximal regulatory region of either TAH-circRNAs or other non-TAH-circRNAs in indicated cell lines. **P-value < 0.05.

DNA methylation plays a fundamental role in regulating gene expression, and it is well-established that an inverse relationship exists between DNA methylation and transcriptional activity at cis-regulatory regions, such as enhancers and promoters (Lister et al., 2009; Silva et al., 2019). CircRNAs with hypo-methylated regulatory regions were occupied by more TFs than those with hyper-methylated regions across multiple cell lines, indicative of more active TF regulation (Figures 3A,B and Supplementary Figures 3A,B). Notably, DNA methylation was significantly lower in regulatory regions of TAH-circRNAs relative to other circRNAs (Figure 3C and Supplementary Figure 3C), suggesting that the hypo-methylated regulatory regions in TAH-circRNAs are more accessible for TF binding and regulation. This methylation pattern is consistent with our earlier characterizations of other epigenomic features of TAH-circRNAs, displaying more accessible regulatory regions, stronger transcriptional activities, as well as higher expression levels.

### Comprehensive Analysis of TF-CircRNA Regulatory Network

**Figure 4:**
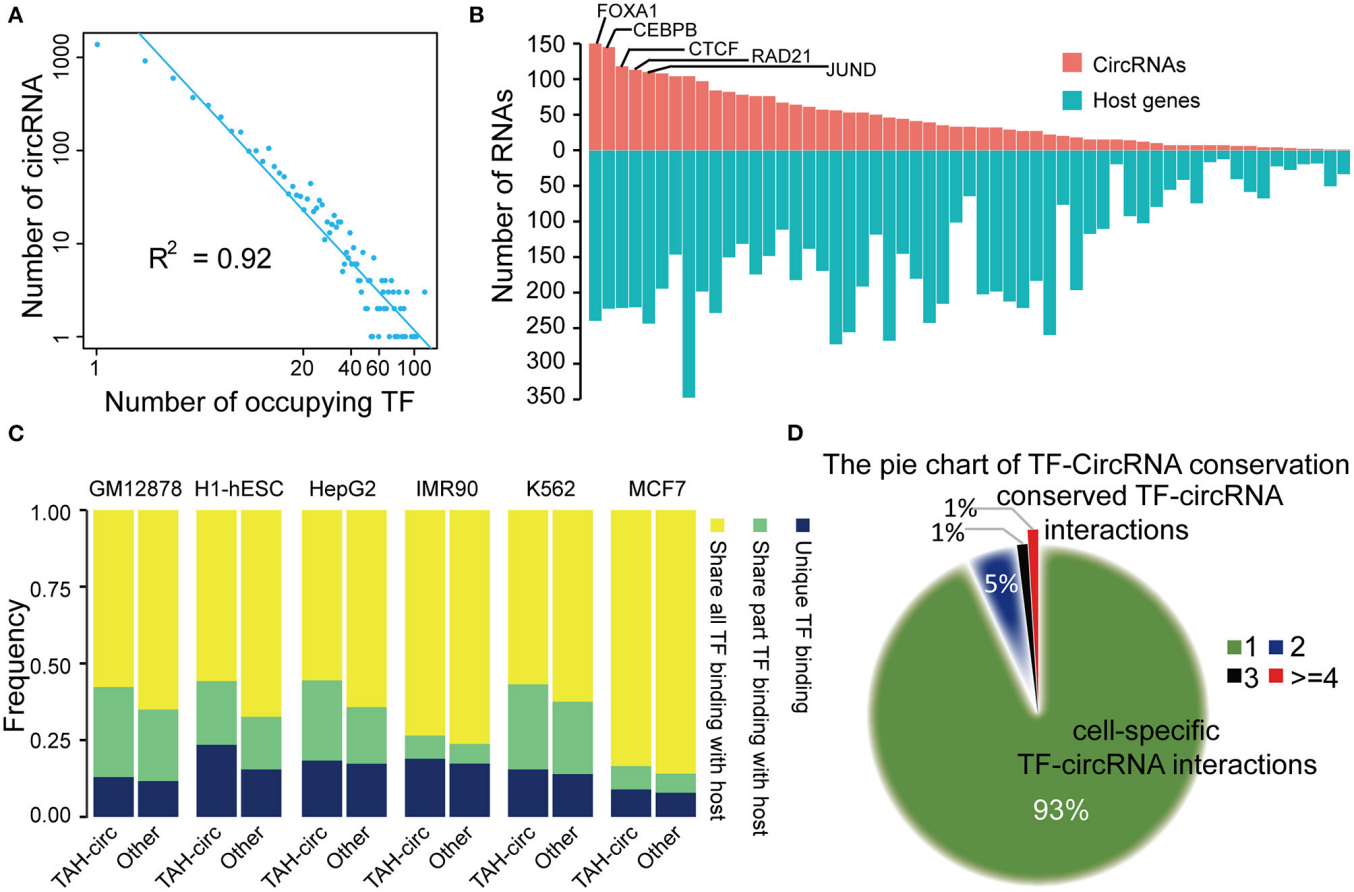
Analysis of TF-circRNAs regulatory network. **(A)** Degree distribution of TAH-circRNAs occupied by the increased number of TFs. X axis, the number of occupying TFs; Y axis, the number of TAH-circRNAs. **(B)** The rank of TFs ordered by the number of circRNAs (red bars) or host genes (blue bars) they occupy in HepG2 cells. **(C)** The proportion of TAH-circRNAs that share TF binding with their host genes in indicated cell lines. Blue, TAH-circRNAs do not share TFs binding with corresponding host genes; green, TAH-circRNAs share part TFs binding with host genes; yellow, TAH-circRNAs share all TFs binding with host genes. **(D)** Pie chart showing the distribution of TF-circRNA regulatory relationships across different cell lines.

To further understand the transcriptional regulatory mechanisms of TAH-circRNA, we identified TFs that had binding sites within the potential proximal regulatory region of TAH-circRNAs (between 2 Kb upstream and 1 Kb downstream of the TSS) by analyzing 159 TF ChIP-seq results from the matched six cell lines (ENCODE database). We first analyzed the distribution degree of TF-circRNAs regulatory network, and determined that it was power law distribution (Figure 4A and Supplementary Figure 4A). Because the transcriptional regulation network of coding genes is also strictly following power law distribution, this result suggests that TAH-circRNAs share similar transcription mechanism by with coding genes.

Again highly similar as the transcriptional pattern of coding genes (Hnisz et al., 2015; Peeters et al., 2015; Saint-Andre et al., 2016), some of the circRNAs were occupied by a large number of TFs (lower right corner of Figure 4A). It has been well-established that a gene (often termed “Hub gene”) with more interacting partners or sharing more upstream TFs with other genes is potentially more significant functionally in relevant biological processes (Arnone and Davidson, 1997; Wagner, 1999; Odom et al., 2006). Thus, these “Hub circRNAs” may likewise have important functions.

We observed that top-ranked TFs occupying TAH-circRNAs were similar with top-ranked TFs occupying coding genes (Figure 4B and Supplementary Figures 4C–E), suggesting that some master TFs have similar significance for regulation of host genes and circRNAs. Although a number of circRNAs share TFs with their host genes, some TFs were found to only bind to the regulatory region of circRNAs, but not that of their host genes (between 2 Kb upstream and 1 Kb downstream of the host TSS) (Figure 4C). Moreover, relative to other circRNAs, the regulatory regions of TAH-circRNAs had more unique TF binding (Figure 4C). The result imply that a proportion of TAH-circRNAs may be regulated by certain specific TFs independent of host genes.

We found that ~93% TF-circRNA regulatory relationships occurred in only one out of six cell lines (Figure 4D) and only ~1% such regulatory relationships were identified in more than four cell lines. This distribution pattern is again highly consistent with the regulation pattern between TF-coding gene, which has strong cell type- and lineage-specificity (Barshir et al., 2014).

**Figure 5:**
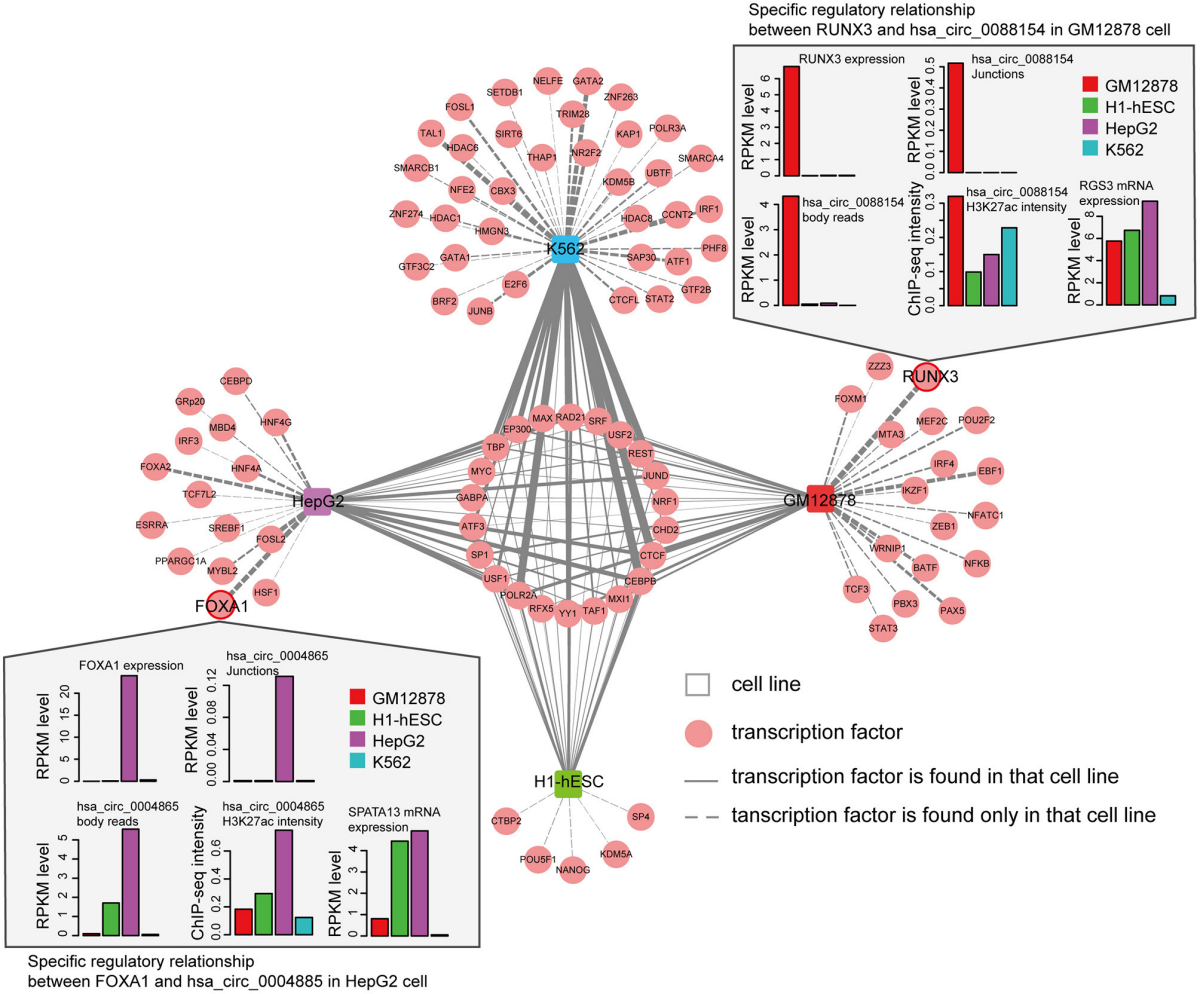
Cell-type-specific TF-circRNA regulatory network. Cell-type-specific (outside, connected by dashed lines) and shared (inside, connected by solid lines) TFs were identified by ChIP-seq data in indicated cell lines. The thickness of the line is proportional to the degree of TF in TF-circRNA regulatory network. Upper and lower insets show two examples of expression profiles of cell-type-specific TFs, host genes, and circRNAs.

We next asked whether circRNAs occupied by cell-type specific TFs are preferentially expressed in corresponding cells. Since the majority of the ENCODE ChIP-seq data was produced from four cell lines (K562, GM12878, HepG2, and H1-hESC), this analysis was restricted to these four cells. We highlighted several TFs and circRNAs that were highly expressed in a particular cell line. For example, we found that RUNX3 specially bound to the proximal regulatory region of hsa_circ_0088154 in GM12878 cells, and both RUNX3 and hsa_circ_0088154 were only expressed in GM12878 cells (Figure 5). Moreover, proximal regulatory region of hsa_circ_0088154 had more H3K27ac signals in GM12878 than in the other three cell lines (top right inset in Figure 5). In contrast, RGS3, the host gene of hsa_circ_0088154, had a different and inconsistent expression pattern (top right inset in Figure 5). As another example, we found that FOXA1 interacted with the hsa_circ_0004865 only in HepG2 cell line (FOXA1 and hsa_circ_004865 are shown in the lower left inset of Figure 5). FOXA1 and hsa_circ_0004865, but not its host gene SPTA13, were only highly expressed in HepG2 cells (Supplementary Figure 4B). These results suggest that TAH-circRNAs are under the regulation of specific TFs in a cell type-specific manner.

### Characterization of SE-Assigned CircRNAs

**Figure 6:**
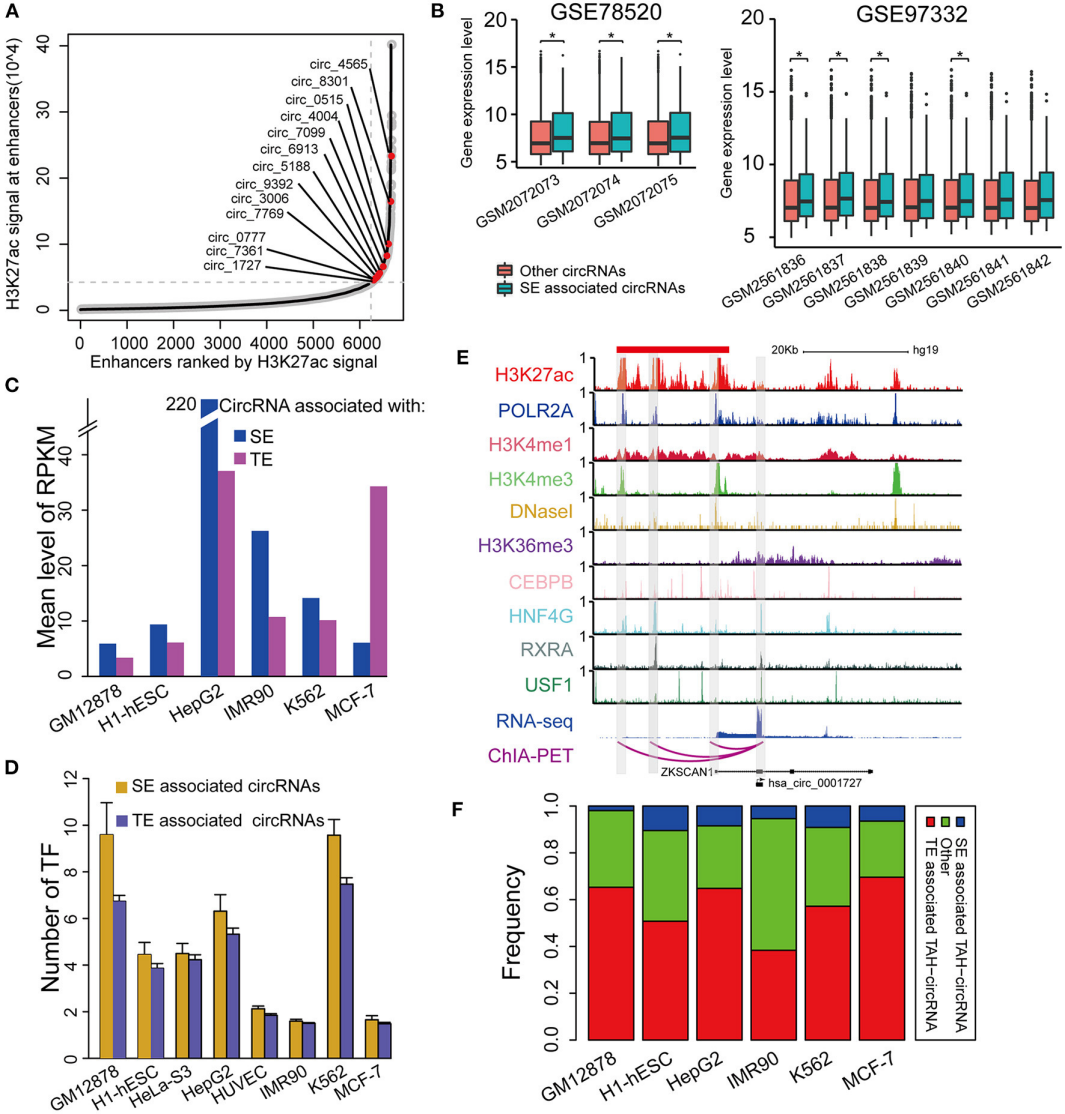
Identification of SE associated circRNAs. **(A)** Hockeystick of the distribution of H3K27ac ChIP-seq signals in HepG2 cell line. SEs are annotated as the enhancers above the inflection point of the curve. **(B)** Box plots of the mean expression of circRNAs associated with SEs from two different cohorts of HCC samples (one-sided Wilcoxon rank-sum test, *P < 0.05). **(C)** Mean expression level of SE- and TE-associated circRNAs in indicated cell lines. **(D)** The number of TFs binding to SE- and TE-associated circRNAs in indicated cell lines. **(E)** IGV plots showing ChIP-seq profiles for indicated TFs and histone marks of hsa_circ_0001727 and its host gene ZKSCAN1 in HepG2 cells. RNA-seq and ChIA-PET tracks are shown in the bottom. **(F)** The fractions of TAH-circRNAs that were associated with either SEs or TEs.

Super-enhancers (SEs) play key regulatory roles in transcriptional activation in both normal cell development and disease states (Hnisz et al., 2013; Loven et al., 2013; Jiang et al., 2019; Qian et al., 2019; Bai et al., 2020). Very recently, Bin et al. identified an SE-regulated circRNAs (circNfix), which has important functions in cardiomyocyte proliferation and angiogenesis (Huang et al., 2019). However, it is unknown if this report merely represents a particular case. We thus next systematically analyzed the role of SEs in the transcriptional regulation of circRNAs. Importantly, we found that SE-associated circRNAs were up-regulated compared with TE-associated circRNAs in all analyzed cell lines (Figures 6A,C).

Furthermore, the expression levels of SE-associated circRNAs were higher than other circRNAs in HCC clinical samples (one-sided wilcoxon rank-sum test, *P*-value < 0.05, Figure 6B). Consistently, the regulatory regions of SE-associated circRNAs have more TF binding (Figure 6D) and higher H3K4me1 signals (Supplementary Figure 5C) than TE-associated circRNAs. We confirmed the expression of randomly selected twelve circRNAs associated with SEs by Real-time PCR and Sanger sequencing (Table 1, Supplementary Figures 5A,B).

**Table 1:** These circRNAs were verified by Real-time PCR and they are associated with SE.

**CircRNA_ID**	**CircRNA_name**	**Chr**	**Start**	**End**	**Fold_change**	**Hostgene**
circ_4565	hsa_circ_0004565	3	149563797	149619949	3.358573	RNF13
circ_8301	hsa_circ_0008301	11	1307231	1317024	5.081821	TOLLIP
circ_0515	hsa_circ_0000515	14	20811305	20811534	2.685618	RPPH1
circ_4004	hsa_circ_0004004	5	172359438	172362313	2.853213	ERGIC1
circ_7099	hsa_circ_0007099	15	89656955	89659752	2.12391	ABHD2
circ_6913	hsa_circ_0006913	1	27087346	27089776	3.229994	ARID1A
circ_5188	hsa_circ_0005188	19	47858295	47858607	6.494322	DHX34
circ_9392	hsa_circ_0049392	19	11226769	11227674	2.137966	LDLR
circ_3006	hsa_circ_0003006	3	58111307	58112489	6.272582	FLNB
circ_7769	hsa_circ_0007769	6	144086397	144086935	3.981757	PHACTR2
circ_0777	hsa_circ_0040777	16	87453388	87454322	2.029921	ZCCHC14
circ_7361	hsa_circ_0007361	16	87451065	87452507	2.094044	ZCCHC14
circ_1727	hsa_circ_0001727	7	99621041	99621930	1.681775	ZKSCAN1

One exemplary SE-associated circRNA (has_circ_0001827) was shown in Figure 6E. A prominent SE cluster (Red bar in Figure 6E) was identified upstream of this circRNA, which also had notable H3K36me3 signals along its gene body, indicative of active transcriptional elongation. Importantly, ChIA-PET results identified that the TSS of has_circ_0001727 had direct DNA-DNA contact with three SE constituents (Shaded regions in Figure 6E). These SE constituents were co-occupied by several TFs, including POLR2A, HNF4G, CEBPB, RXRA, and USF1. Moreover, the proximal regulatory region of this circRNA was directly occupied by RXRA and USF1. Lastly, compared with its host gene, has_circ_0001727 had much higher expression levels (>17-fold). These results together strongly suggest that has_circ_0001727 is transcriptionally activated by several TFs via its upstream SE region. In addition, we found that 38–70% TAH-circRNAs were associated with TEs, and 2–10% among these circRNAs were associated with SE (Figure 6F).

### Identification and Validation of TAH-CircRNAs Transcriptionally Regulated by FOXA1

Our earlier results (Figure 4B) showed that FOXA1 had the highest number of binding sites with in regulatory regions of TAH-circRNAs. We thus next sought to functionally validate the transcriptional regulation on TAH-circRNAs, using FOXA1 as a model TF.

**Figure 7:**
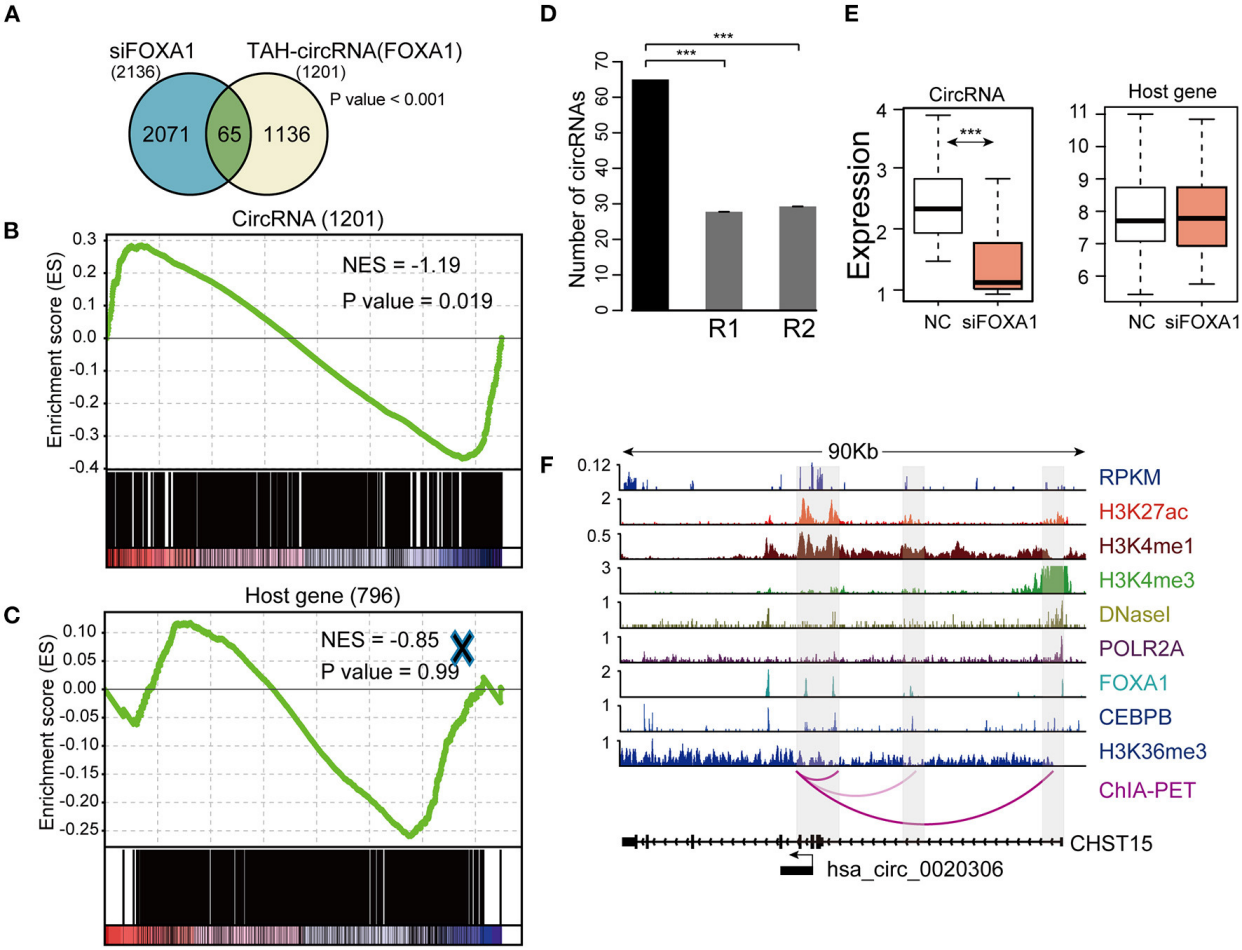
Identification and validation of TAH-circRNAs transcriptionally regulated by FOXA1. **(A)** Venn diagram showing the overlap between the down-regulated circRNAs after FOXA1 knockdown (Left) and circRNAs bound by FOXA1 (Right), *P*-value was performed using hypergeometric test, ***P < 0.001. **(B)** Gene set enrichment analysis of the expression changes of FOXA1-binding TAH-circRNAs and **(C)** their corresponding host genes. **(D)** The number of down-regulated circRNAs overlapping with the TAH-circRNAs that were bound by FOXA1 (black bar) compared with 20,000 random circRNA sets (gray bars). R1: randomly sampling from 92,375 human circRNAs from circBase database. R2: randomly sampling from 19,805 human circRNAs that were bound by FOXA1. **(E)** Expression changes of the 65 FOXA1-binding TAH-circRNA (left) and their corresponding host genes (right) in response to FOXA1 knockdown. NC, negative control. **(F)** IGV plots of RNA-seq and ChIP-seq profiles for the indicated histone marks and TFs for hsa_circ_0020306 and its host gene CHST15 loci in HepG2 cells.

To this end, we silenced FOXA1 using siRNA in HepG2 cells and quantified the genome-wide changes of the expression of both coding genes and circRNAs using microarrays. As a result, we identified a total of 2,136 circRNAs down-regulated (FC < 1.4) following FOXA1 silencing. Importantly, 65 of FOXA1-occupied TAH-circRNAs (total *n* = 1,201) were significantly over-presented in these down-regulated circRNAs (Figure 7A). We further performed permutation test by sampling 20,000 times random circRNA sets and confirmed that the over-presentation of FOXA1-occupied TAH-circRNAs was not by change (Figure 7B). Moreover, for the overlapped 65 circRNAs, the expressions of their host genes were not altered by FOXA1-silencing (Figure 7E). In addition, Gene set enrichment analysis (GSEA) demonstrated that FOXA1-occupied TAH-circRNAs were significantly enriched in the down-regulated transcripts upon knocking down of FOXA1 (Figure 7D). In contrast, this significance was not observed in the host genes of these circRNAs (Figure 7C). Figure 7F shows an example of FOXA1-occupied TAH-circRNA (hsa_circ_0020306) whose expression was decreased following FOXA1 knockdown (Fold change = 0.831). Notably, ChIA-PET identified direct interactions between the TSS of this circRNA and distal regions with both H3K27ac and H3K4me1 signals (indicative of potential enhancers). Moreover, FOXA1 directly occupied both the TSS and distal regions together with other TFs including Pol II, strongly suggesting a direct transcriptional regulation of this circRNA. In contrast, expression of its host gene (CHST15) was up-regulated after knocking down of FOXA1 (1.062-fold). Taken together, these results suggested that a set of TAH-circRNAs are under direct regulation by FOXA1 independent of their host genes.

## Discussion

CircRNAs are increasingly recognized as important players in many biological processes (Legnini et al., 2017; Tang et al., 2019). For example, circRNAs are capable of regulating gene expression by competing for protein and miRNA binding (Hansen et al., 2013; Du et al., 2016). They also compete with linear RNA production and regulate the accumulation of full-length mRNA (Ashwal-Fluss et al., 2014). However, upstream regulatory mechanisms of the transcription of circRNAs are still largely unknown. Here, we performed comprehensive epigenomic investigations of human circRNAs, analyzing transcriptome, cistrome, and DNA methylome data from a total of 91 samples. We identified a group of TAH-circRNAs with distinct epigenomic and biological features, including (i) the regulatory region of TAH-circRNAs had higher signals of H3K36me3 and DNaseI than other circRNAs and, consistently, TAH-circRNAs were occupied by more TFs, suggesting stronger transcriptional activation; (ii), TAH-circRNAs had greater conservation score than other circRNAs, indicating their function significance; (iii) the regulatory regions of TAH-circRNAs are markedly more hypo-methylated than other circRNAs, in agreement with its higher transcriptional output.

Most importantly, we provided a number of lines of evidence to demonstrate that TAH-circRNAs are under direct transcriptional regulations mediated by TFs. First, ChIA-PET results identified that 23–52% of TSS of TAH-circRNAs had direct interactions with distal cis-regulatory regions. In comparison, only 11–46% of non-TAH-circRNAs displayed such contacts. As shown in our exemplary circRNAs (Figures 2E, 6E, 7F), these DNA-DNA interactions almost always occurred between the circRNA TSS and potential enhancer regions (positive of enhancer signals and TF binding), strongly suggesting that these TAH-circRNAs can be directly transcribed independent of host genes. Second, we found a subset of TF-circRNA regulatory pairs that did not involve their host genes (Figure 5). Third, we identified SE-associated circRNAs that had higher expression levels than other circRNAs. Fourth, functional validation further confirmed that FOXA1 directly regulated the transcription of at least 65 TAH-circRNAs independent of their host genes.

In summary, this work systematically analyzes the epigenomic features of circRNAs and identifies a distinct group of TAH-circRNAs. Moreover, our study demonstrates that independent transcriptional mechanisms exist for the regulation of the expression of circRNAs. These results together shed important insights into the regulation of this large class of non-coding RNAs.

## Data Availability Statement

The accession number for the microarray data reported in this paper is NCBI GEO: GSE161174.

## Author Contributions

X-CL and Z-DT analyzed data and wrote the paper. Y-YL, F-CQ, J-MZ, ML, JZha, X-FB, JZhu, and C-CF participated in the pre-processing of the datasets and performed the computational analysis. LP, L-WD, and X-JD performed the experiment validation. Q-YW, JP, and C-QL conceived the idea for the paper, provided the guidance, and critically revised the paper. All authors read and approved the final version to be published.

## Conflict of Interest

The authors declare that the research was conducted in the absence of any commercial or financial relationships that could be construed as a potential conflict of interest.

## References

[B1] Arnone M. I. Davidson E. H. (1997). The hardwiring of development: organization and function of genomic regulatory systems. Development 124, 1851–1864. 9169833 10.1242/dev.124.10.1851

[B2] Ashwal-Fluss R. Meyer M. Pamudurti N. R. Ivanov A. Bartok O. Hanan M.. (2014). circRNA biogenesis competes with pre-mRNA splicing. Mol. Cell 56, 55–66. 10.1016/j.molcel.2014.08.019 25242144

[B3] Bai X. Shi S. Ai B. Jiang Y. Liu Y. Han X.. (2020). ENdb: a manually curated database of experimentally supported enhancers for human and mouse. Nucleic Acids Res. 48, D51–D57. 10.1093/nar/gkz973 31665430 PMC7145688

[B4] Barshir R. Shwartz O. Smoly I. Y. Yeger-Lotem E. (2014). Comparative analysis of human tissue interactomes reveals factors leading to tissue-specific manifestation of hereditary diseases. PLoS Comput. Biol. 10:e1003632. 10.1371/journal.pcbi.1003632 24921629 PMC4055280

[B5] Chen L. L. (2016). The biogenesis and emerging roles of circular RNAs. Nat. Rev. Mol. Cell Biol. 17, 205–211. 10.1038/nrm.2015.32 26908011

[B6] Dong R. Ma X. K. Chen L. L. Yang L. (2017). Increased complexity of circRNA expression during species evolution. RNA Biol. 14, 1064–1074. 10.1080/15476286.2016.1269999 27982734 PMC5680680

[B7] Du W. W. Yang W. Liu E. Yang Z. Dhaliwal P. Yang B. B. (2016). Foxo3 circular RNA retards cell cycle progression via forming ternary complexes with p21 and CDK2. Nucleic Acids Res. 44, 2846–2858. 10.1093/nar/gkw027 26861625 PMC4824104

[B8] Feng C. Song C. Liu Y. Qian F. Gao Y. Ning Z.. (2020). KnockTF: a comprehensive human gene expression profile database with knockdown/knockout of transcription factors. Nucleic Acids Res. 48, D93–D100. 10.1093/nar/gkz881 31598675 PMC6943067

[B9] Hansen T. B. Jensen T. I. Clausen B. H. Bramsen J. B. Finsen B. Damgaard C. K.. (2013). Natural RNA circles function as efficient microRNA sponges. Nature 495, 384–388. 10.1038/nature11993 23446346

[B10] Hnisz D. Abraham B. J. Lee T. I. Lau A. Saint-Andre V. Sigova A. A.. (2013). Super-enhancers in the control of cell identity and disease. Cell 155, 934–947. 10.1016/j.cell.2013.09.053 24119843 PMC3841062

[B11] Hnisz D. Schuijers J. Lin C. Y. Weintraub A. S. Abraham B. J. Lee T. I.. (2015). Convergence of developmental and oncogenic signaling pathways at transcriptional super-enhancers. Mol. Cell 58, 362–370. 10.1016/j.molcel.2015.02.014 25801169 PMC4402134

[B12] Hsiao K. Y. Lin Y. C. Gupta S. K. Chang N. Yen L. Sun H. S.. (2017). Noncoding effects of circular RNA CCDC66 promote colon cancer growth and metastasis. Cancer Res. 77, 2339–2350. 10.1158/0008-5472.CAN-16-1883 28249903 PMC5910173

[B13] Huang S. Li X. Zheng H. Si X. Li B. Wei G.. (2019). Loss of super-enhancer-regulated circRNA Nfix induces cardiac regeneration after myocardial infarction in adult mice. Circulation 139, 2857–2876. 10.1161/CIRCULATIONAHA.118.038361 30947518 PMC6629176

[B14] Jiang Y. Qian F. Bai X. Liu Y. Wang Q. Ai B.. (2019). SEdb: a comprehensive human super-enhancer database. Nucleic Acids Res. 47, D235–D243. 10.1093/nar/gky1025 30371817 PMC6323980

[B15] Kharchenko P. V. Tolstorukov M. Y. Park P. J. (2008). Design and analysis of ChIP-seq experiments for DNA-binding proteins. Nat. Biotechnol. 26, 1351–1359. 10.1038/nbt.1508 19029915 PMC2597701

[B16] Kristensen L. S. Okholm T. L. H. Veno M. T. Kjems J. (2018). Circular RNAs are abundantly expressed and upregulated during human epidermal stem cell differentiation. RNA Biol. 15, 280–291. 10.1080/15476286.2017.1409931 29283313 PMC5798954

[B17] Legnini I. Di Timoteo G. Rossi F. Morlando M. Briganti F. Sthandier O.. (2017). Circ-ZNF609 is a circular RNA that can be translated and functions in myogenesis. Mol. Cell 66, 22–37 e9. 10.1016/j.molcel.2017.02.017 28344082 PMC5387670

[B18] Li G. Chen Y. Snyder M. P. Zhang M. Q. (2017). ChIA-PET2: a versatile and flexible pipeline for ChIA-PET data analysis. Nucleic Acids Res. 45:e4. 10.1093/nar/gkw809 27625391 PMC5224499

[B19] Li X. Liu C. X. Xue W. Zhang Y. Jiang S. Yin Q. F.. (2017). Coordinated circRNA biogenesis and function with NF90/NF110 in viral infection. Mol. Cell 67, 214–227 e7. 10.1016/j.molcel.2017.05.023 28625552

[B20] Li Y. Li X. Yang Y. Li M. Qian F. Tang Z. (2020). TRlnc: a comprehensive database for human transcriptional regulatory information of lncRNAs. Brief. Bioinform. 10.1093/bib/bbaa011. [Epub ahead of print]. 32047897

[B21] Lister R. Pelizzola M. Dowen R. H. Hawkins R. D. Hon G. Tonti-Filippini J.. (2009). Human DNA methylomes at base resolution show widespread epigenomic differences. Nature 462, 315–322. 10.1038/nature08514 19829295 PMC2857523

[B22] Loven J. Hoke H. A. Lin C. Y. Lau A. Orlando D. A. Vakoc C. R.. (2013). Selective inhibition of tumor oncogenes by disruption of super-enhancers. Cell 153, 320–334. 10.1016/j.cell.2013.03.036 23582323 PMC3760967

[B23] McGinnis S. Madden T. L. (2004). BLAST: at the core of a powerful and diverse set of sequence analysis tools. Nucleic Acids Res. 32, W20–W25. 10.1093/nar/gkh435 15215342 PMC441573

[B24] Memczak S. Jens M. Elefsinioti A. Torti F. Krueger J. Rybak A.. (2013). Circular RNAs are a large class of animal RNAs with regulatory potency. Nature 495, 333–338. 10.1038/nature11928 23446348

[B25] Meng J. Chen S. Han J. X. Qian B. Wang X. R. Zhong W. L.. (2018). Twist1 regulates vimentin through Cul2 circular RNA to promote EMT in hepatocellular carcinoma. Cancer Res. 78, 4150–4162. 10.1158/0008-5472.CAN-17-3009 29844124

[B26] Odom D. T. Dowell R. D. Jacobsen E. S. Nekludova L. Rolfe P. A. Danford T. W.. (2006). Core transcriptional regulatory circuitry in human hepatocytes. Mol. Syst. Biol. 2:200602017. 10.1038/msb4100059 16738562 PMC1681491

[B27] Peeters J. G. Vervoort S. J. Tan S. C. Mijnheer G. de Roock S. Vastert S. J.. (2015). Inhibition of super-enhancer activity in autoinflammatory site-derived T cells reduces disease-associated gene expression. Cell Rep. 12, 1986–1996. 10.1016/j.celrep.2015.08.046 26387944

[B28] Qian F. C. Li X. C. Guo J. C. Zhao J. M. Li Y. Y. Tang Z. D.. (2019). SEanalysis: a web tool for super-enhancer associated regulatory analysis. Nucleic Acids Res. 47, W248–W55. 10.1093/nar/gkz302 31028388 PMC6602466

[B29] Rybak-Wolf A. Stottmeister C. Glazar P. Jens M. Pino N. Giusti S.. (2015). Circular RNAs in the mammalian brain are highly abundant, conserved, and dynamically expressed. Mol. Cell 58, 870–885. 10.1016/j.molcel.2015.03.027 25921068

[B30] Saint-Andre V. Federation A. J. Lin C. Y. Abraham B. J. Reddy J. Lee T. I.. (2016). Models of human core transcriptional regulatory circuitries. Genome Res. 26, 385–396. 10.1101/gr.197590.115 26843070 PMC4772020

[B31] Salzman J. Chen R. E. Olsen M. N. Wang P. L. Brown P. O. (2013). Cell-type specific features of circular RNA expression. PLoS Genet. 9:e1003777. 10.1371/annotation/f782282b-eefa-4c8d-985c-b1484e845855 24039610 PMC3764148

[B32] Silva T. C. Coetzee S. G. Gull N. Yao L. Hazelett D. J. Noushmehr H.. (2019). ELMER v.2: an R/Bioconductor package to reconstruct gene regulatory networks from DNA methylation and transcriptome profiles. Bioinformatics 35, 1974–1977. 10.1093/bioinformatics/bty902 30364927 PMC6546131

[B33] Tang Z. Li X. Zhao J. Qian F. Feng C. Li Y.. (2019). TRCirc: a resource for transcriptional regulation information of circRNAs. Brief. Bioinform. 20, 2327–2333. 10.1093/bib/bby083 30184150

[B34] Thorvaldsdottir H. Robinson J. T. Mesirov J. P. (2013). Integrative genomics viewer (IGV): high-performance genomics data visualization and exploration. Brief. Bioinform. 14, 178–192. 10.1093/bib/bbs017 22517427 PMC3603213

[B35] Veno M. T. Hansen T. B. Veno S. T. Clausen B. H. Grebing M. Finsen B.. (2015). Spatio-temporal regulation of circular RNA expression during porcine embryonic brain development. Genome Biol. 16:245. 10.1186/s13059-015-0801-3 26541409 PMC4635978

[B36] Wagner A. (1999). Genes regulated cooperatively by one or more transcription factors and their identification in whole eukaryotic genomes. Bioinformatics 15, 776–784. 10.1093/bioinformatics/15.10.776 10705431

[B37] Westholm J. O. Miura P. Olson S. Shenker S. Joseph B. Sanfilippo P.. (2014). Genome-wide analysis of drosophila circular RNAs reveals their structural and sequence properties and age-dependent neural accumulation. Cell Rep. 9, 1966–1980. 10.1016/j.celrep.2014.10.062 25544350 PMC4279448

[B38] Yao Z. Luo J. Hu K. Lin J. Huang H. Wang Q.. (2017). ZKSCAN1 gene and its related circular RNA (circZKSCAN1) both inhibit hepatocellular carcinoma cell growth, migration, and invasion but through different signaling pathways. Mol. Oncol. 11, 422–437. 10.1002/1878-0261.12045 28211215 PMC5527481

[B39] You X. Vlatkovic I. Babic A. Will T. Epstein I. Tushev G.. (2015). Neural circular RNAs are derived from synaptic genes and regulated by development and plasticity. Nat. Neurosci. 18, 603–610. 10.1038/nn.3975 25714049 PMC4376664

[B40] Zhang X. O. Dong R. Zhang Y. Zhang J. L. Luo Z. Zhang J.. (2016). Diverse alternative back-splicing and alternative splicing landscape of circular RNAs. Genome Res. 26, 1277–1287. 10.1101/gr.202895.115 27365365 PMC5052039

[B41] Zhang Y. Liu T. Meyer C. A. Eeckhoute J. Johnson D. S. Bernstein B. E.. (2008). Model-based analysis of ChIP-Seq (MACS). Genome Biol. 9:R137. 10.1186/gb-2008-9-9-r137 18798982 PMC2592715

[B42] Zhang Y. Xue W. Li X. Zhang J. Chen S. Zhang J. L.. (2016). The biogenesis of nascent circular RNAs. Cell Rep. 15, 611–624. 10.1016/j.celrep.2016.03.058 27068474

[B43] Zhou C. Liu H. S. Wang F. W. Hu T. Liang Z. X. Lan N.. (2020). circCAMSAP1 promotes tumor growth in colorectal cancer via the miR-328-5p/E2F1 axis. Mol. Ther. 28, 914–928. 10.1016/j.ymthe.2019.12.008 31951832 PMC7054739

