# Supplementary material for: Integrative Epigenomic Analysis of Transcriptional Regulation of Human CircRNAs
Source: Front Genet. 2021 Jan 25;11:590672. doi: 10.3389/fgene.2020.590672 (PMC7868561; doi:10.3389/fgene.2020.590672)
Supplement: Supplementary file 1 [file Data_Sheet_1.docx]

**Supplementary Figure Legends**

**Supplementary Figure 1. Expression analysis of circRNAs in various cell lines.** (A-F) Distribution of uniquely identified circRNA (left) and total circRNA (right) in indicated cell lines. X axis, the RPKM value of circRNAs; Y axis, the RPKM value of host genes. (G) Pie chart showing the distribution of uniquely identified circRNAs that have higher or lower RPKM than host genes in indicated cell lines. (H) Pie chart showing the distribution of uniquely identified circRNAs that have higher or lower H3K27ac signal than host gene in indicated cell lines.


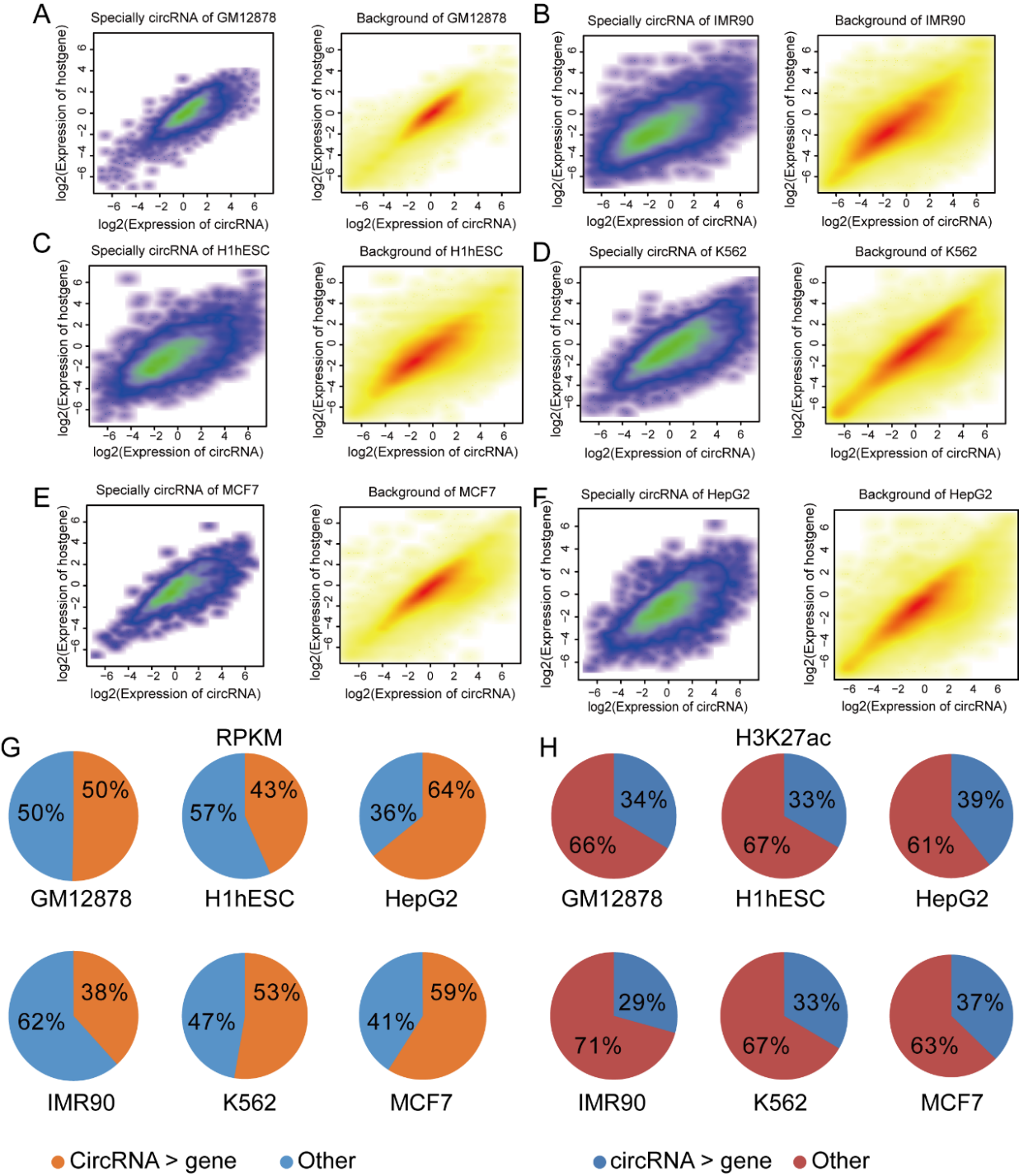


**Supplementary Figure 2. Epigenomic analyses of TAH-circRNAs in indicated human cell lines.** (A) Pie chart showing the distribution of uniquely identified circRNAs that have higher or lower RPKM than host gene in indicated cell lines. Metagenes representation of indicated ChIP-seq profiles including H3K27ac (B), DNaseI (C), H3K4me1 (D), H3K36me3 (E) and pol2 (F) at TAH-circRNAs or other circRNAs in indicated cell lines.


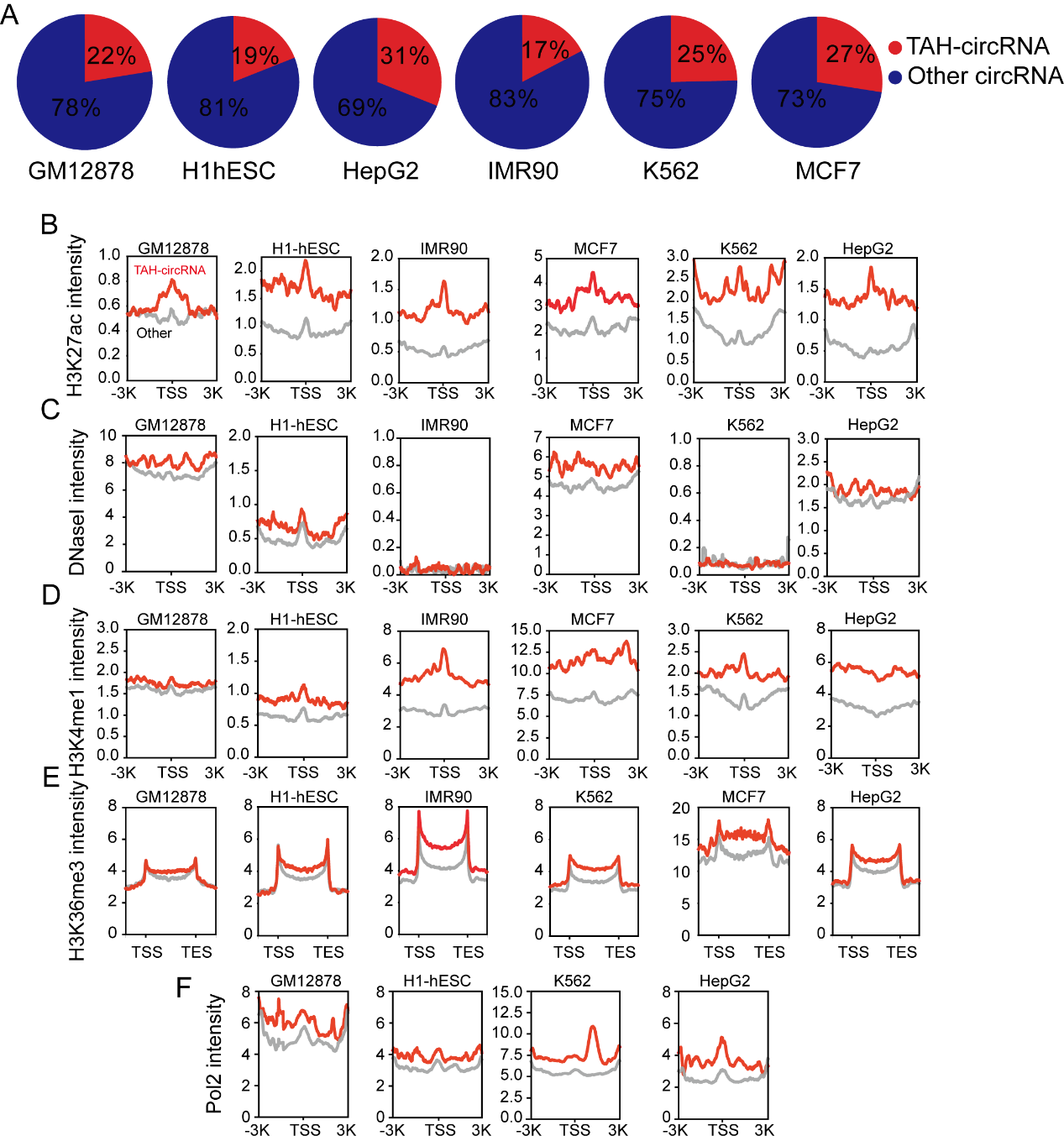


**Supplementary Figure 3. DNA Methylation pattern of circRNAs.** (A) The number of TFs occupying circRNAs in indicated cell lines stratified by the DNA methylation level of the proximal regulatory region of circRNAs using Illumina Infinium Human Methylation 450 Bead Array (450K) data. (B) Mean number of TFs that occupy high and low methylated circRNAs across various cell lines. (C) Level of methylation of TAH-circRNAs in GM12878, H1-hESC, HepG2 and K562 cells (P_value < 0.05).


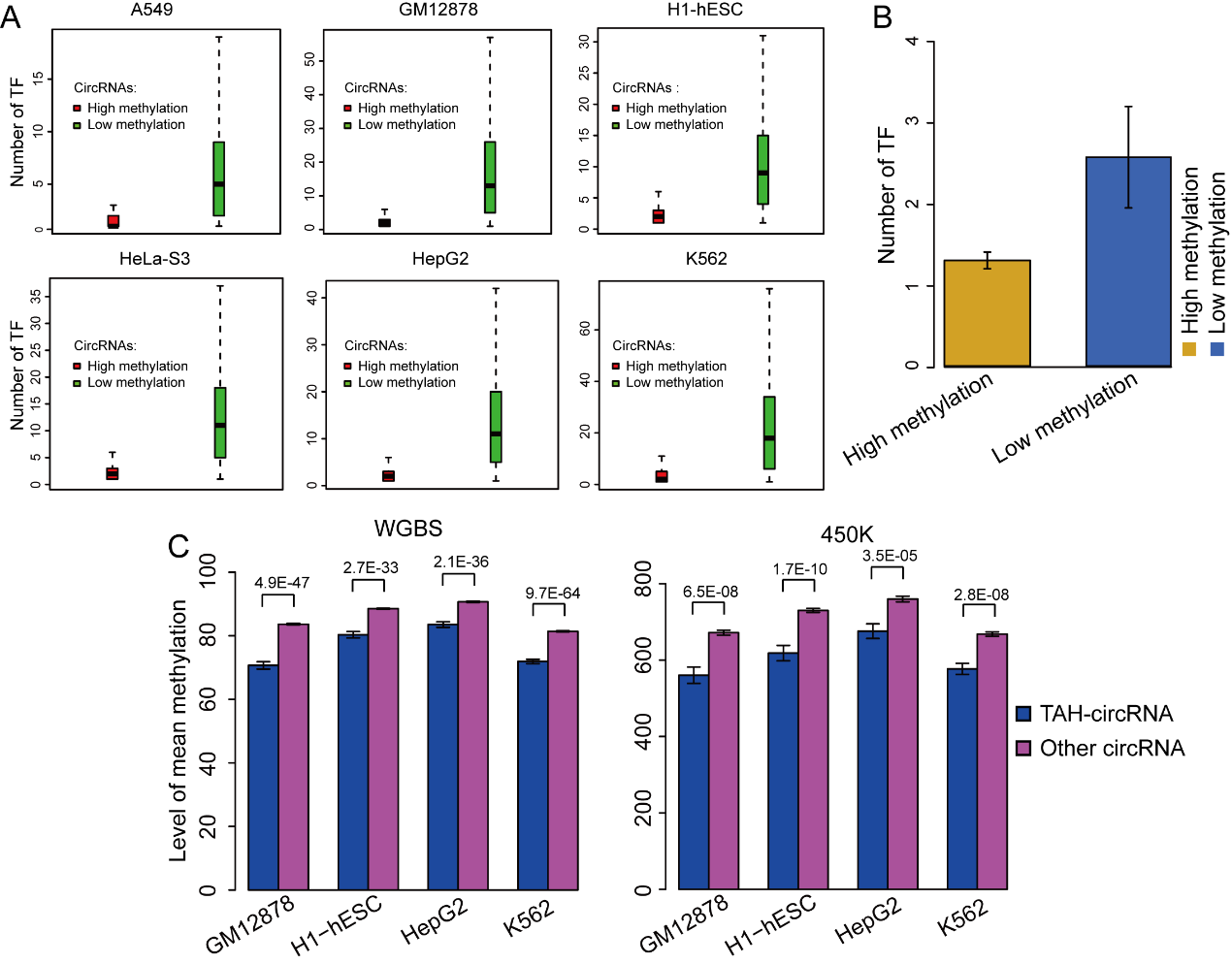


**Supplementary Figure 4. Analysis of TF-circRNAs regulatory network.** (A) Degree distribution of TAH-circRNAs occupied by the increased number of TFs in indicated cell lines. X axis, the number of occupying TFs. Y axis, the number of TAH-circRNAs. (B) Mean expression of circRNAs that are occupied by RUNX3, POU5F1, FOXA1 and TAL1 in GM12878, H1-hESC, HepG2 and K562, respectively. The average number of TAH-circRNAs (left) and coding genes (right) regulated by different TFs in (C) GM12878, (D) H1-hESC and (E) K562 cells.


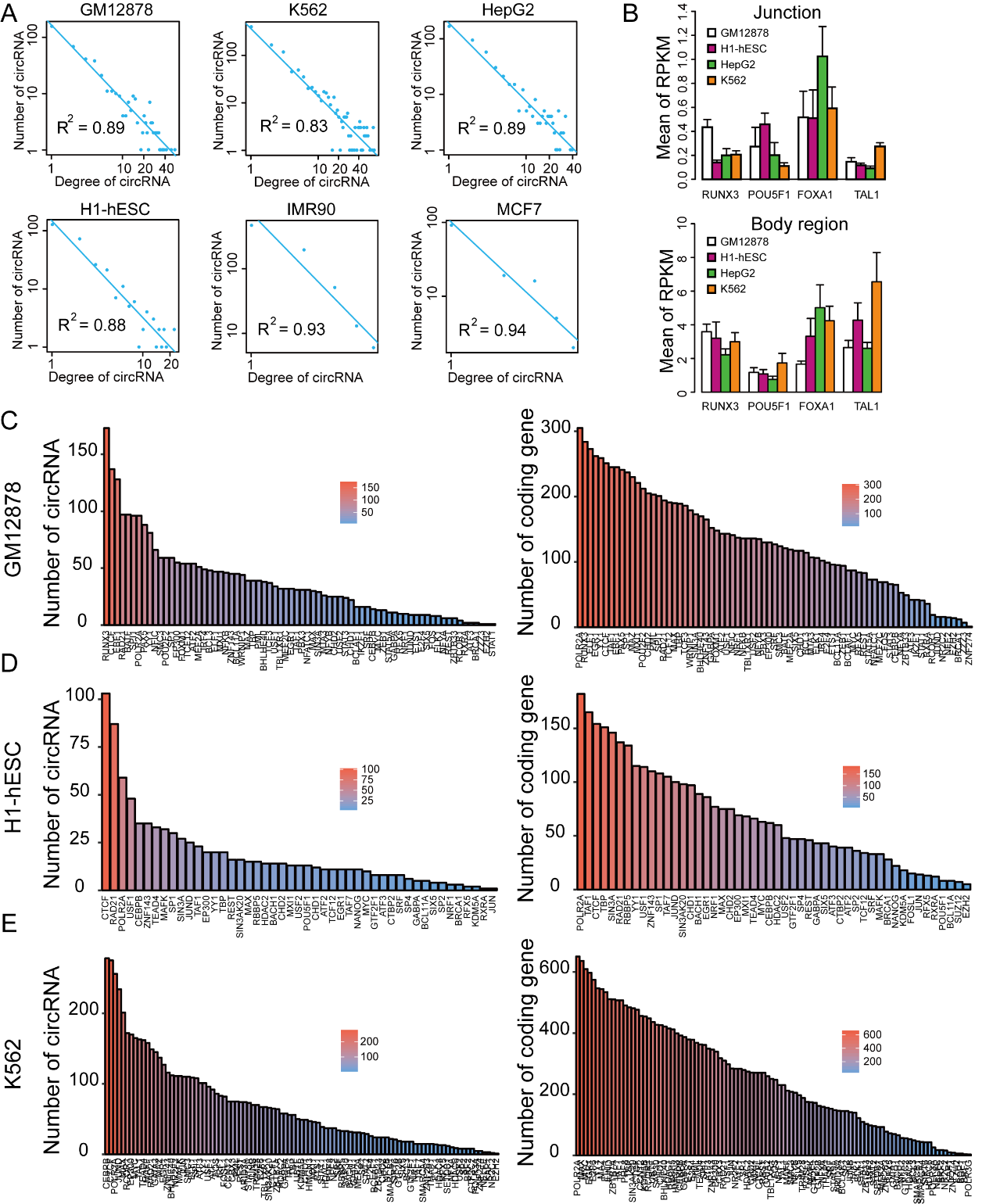


**Supplementary Figure 5. Analyses of SE associated circRNAs.** (A) CircRNAs were measured by Real-time RT-PCR. (B) Electropherograms showing the circular RNA-specific splice junction detected in RT-PCR products for human hsa_circ_0005188, hsa_circ_0008301, hsa_circ_0007769, hsa_circ_0004004 and hsa_circ_0007099. The splice junction is indicated by a black bar. (C) Metagenes representation of ChIP-seq occupancy (H3K4me1) at SE- and TE-associated circRNAs in indicated cell lines. (D) The expression level of cell-type specific super-enhancer related circRNAs in six cell lines. The super-enhancer related circRNAs are described in box plot marked in yellow.


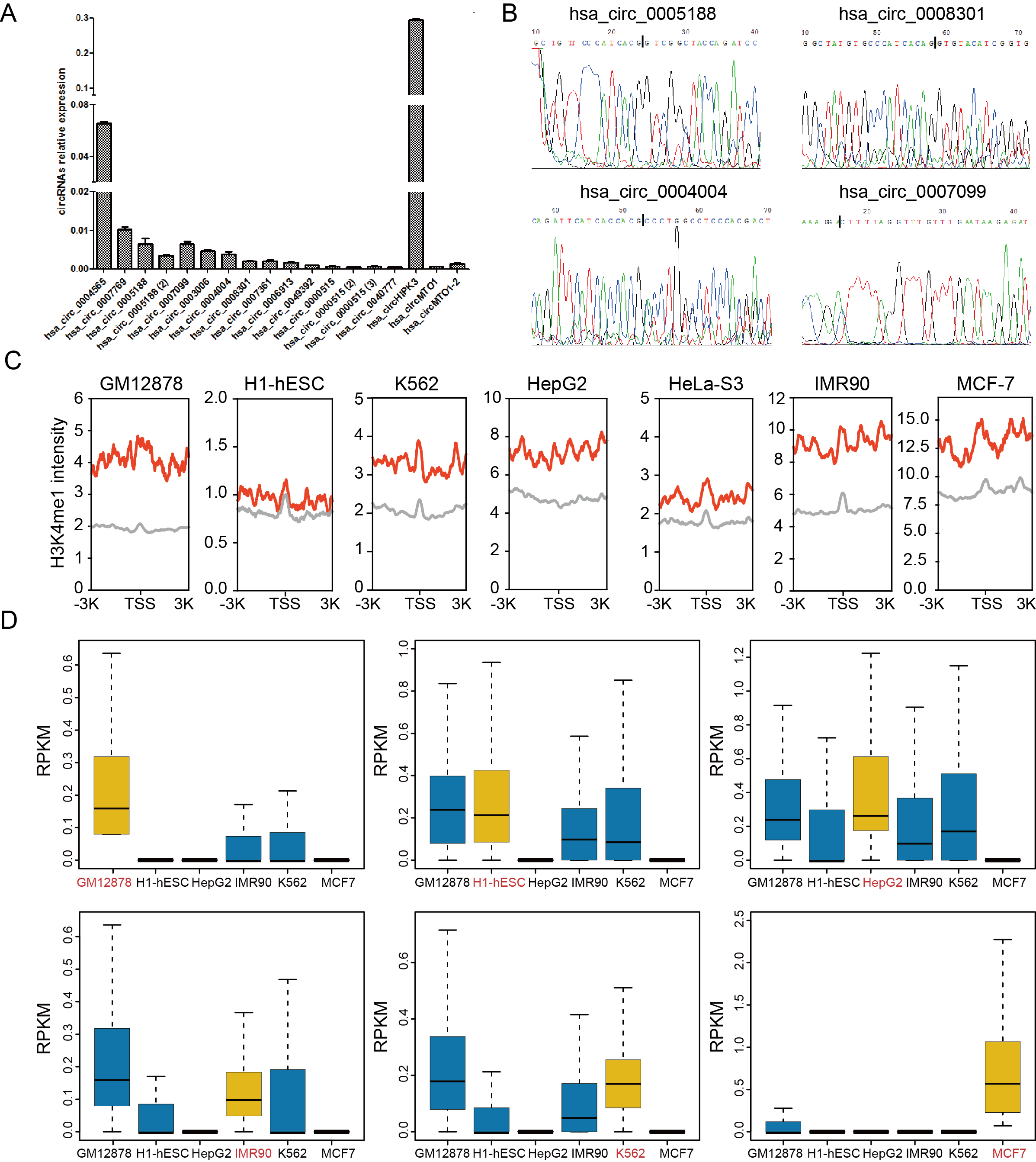


**Supplementary Figure 6. Epigenomic profiles exemplary** **TAH-circRNAs.** RNA-seq, ChIA-PET and ChIP-seq profiles of the indicated histone marks and TFs for hsa_circ_0046294 (A), hsa_circ_0001700 (B), has_circ_0007624 (C), hsa_circ_0061179 (D) and their corresponding host genes.


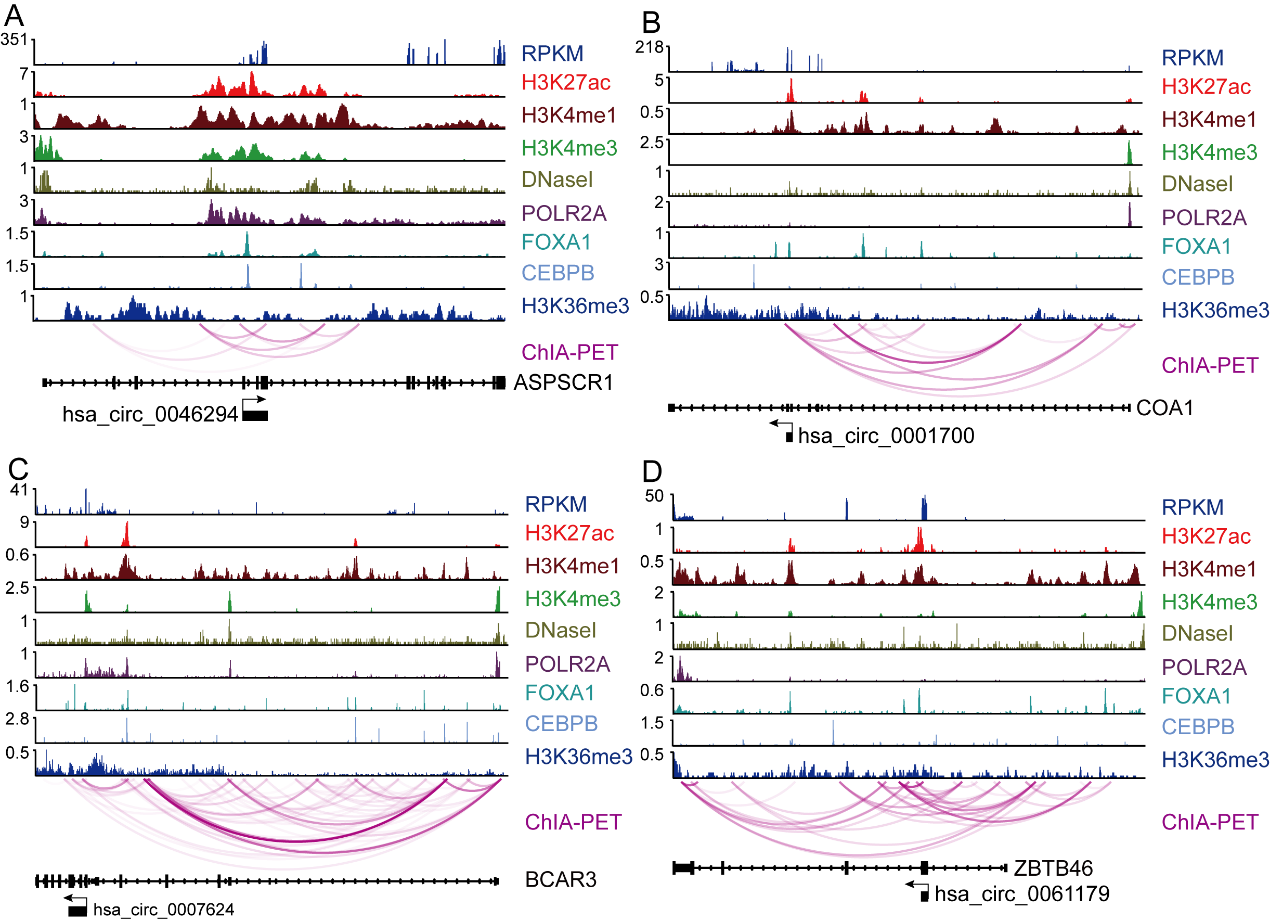


**Supplementary Figure 7. The distribution of TAH-circRNA’s exons and host gene’s exons.** (A) Statistics for the count of TAH-circRNAs and host genes, which contain various number of exons in indicated cell lines, x-axis represent exons number, y-axis represent the count of TAH-circRNAs and host genes. (B) The relationships with the exons of their host gene in the case of 0001727 and 0001900 circRNAs.


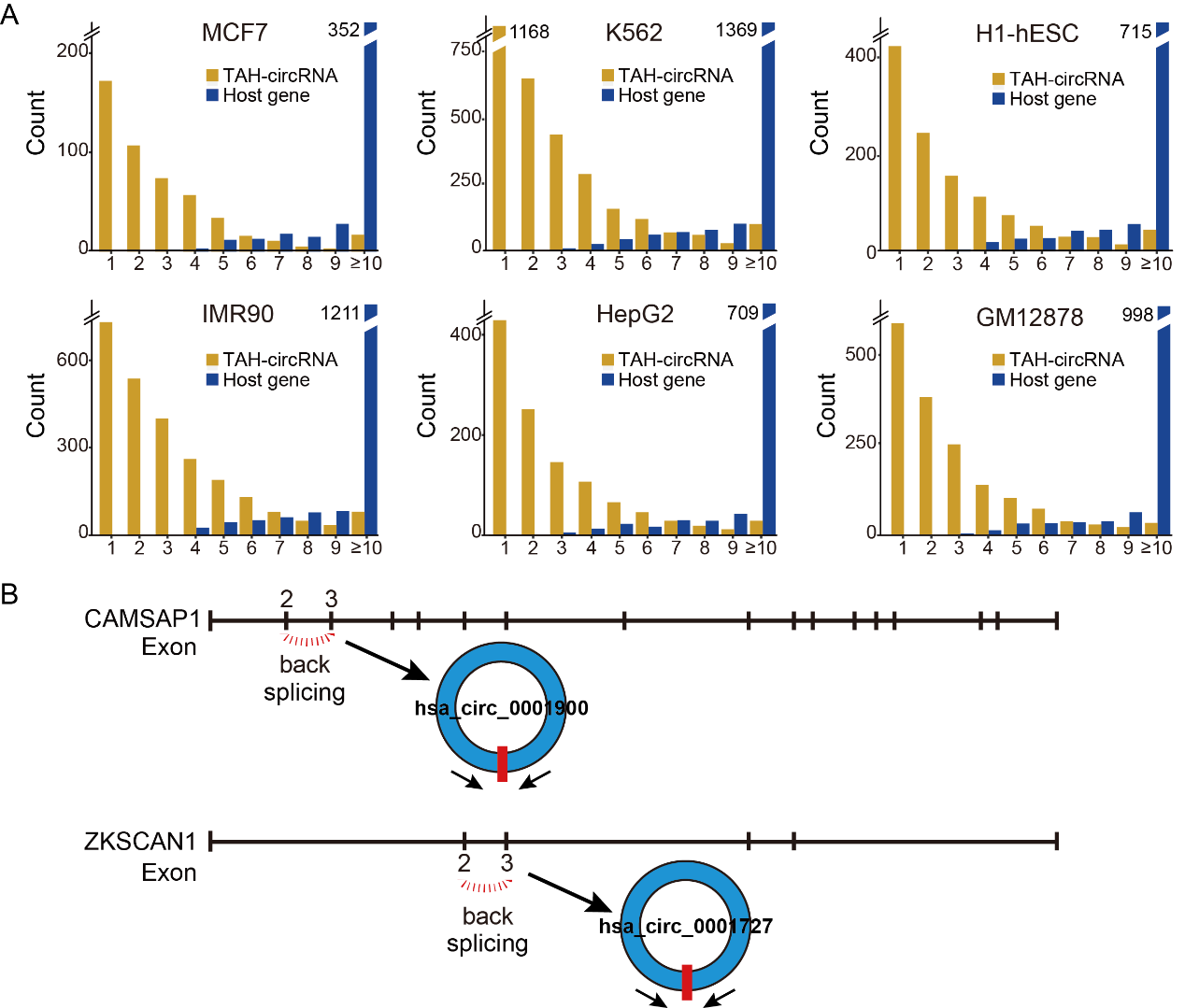


**Supplementary Table Legends**

**Supplementary Table 1. Available uniform TF ChIP-seq, polyA-, total RNA, 450K array, WGBS and H3K27ac data for each cell lines.**

| **Cell_name** | **Uniform TF ChIP-seq** | **polyA-** | **totalRNA** | **450K array** | **WGBS** | **H3K27ac** |
| --- | --- | --- | --- | --- | --- | --- |
| 22Rv1 |  |  |  |  |  | √ |
| A549 | √ | √ |  | √ | √ | √ |
| A673 |  |  |  |  |  | √ |
| ACC112 |  |  |  |  |  | √ |
| AG04449 | √ |  |  | √ |  |  |
| AG04450 | √ | √ |  | √ |  |  |
| AG09309 | √ |  |  | √ |  |  |
| AG09319 | √ |  |  | √ |  |  |
| AG10803 | √ |  |  | √ |  |  |
| AoAF | √ |  |  |  |  |  |
| AoSMC |  |  |  | √ |  |  |
| BE2_C | √ |  |  | √ |  |  |
| BJ | √ | √ |  | √ |  |  |
| C4-2B |  |  |  |  |  | √ |
| Caco-2 | √ |  |  | √ |  |  |
| CMK |  |  |  | √ |  |  |
| Dnd41 | √ |  |  |  |  | √ |
| DOHH2 |  |  |  |  |  | √ |
| ECC-1 | √ |  |  | √ |  |  |
| Fibrobl | √ |  |  | √ |  |  |
| Gliobla | √ |  |  |  |  |  |
| GM06990 | √ |  |  | √ |  |  |
| GM08714 | √ |  |  |  |  |  |
| GM10847 | √ |  |  |  |  |  |
| GM12801 | √ |  |  |  |  |  |
| GM12864 | √ |  |  |  |  |  |
| GM12865 | √ |  |  |  |  |  |
| GM12872 | √ |  |  |  |  |  |
| GM12873 | √ |  |  |  |  |  |
| GM12874 | √ |  |  |  |  |  |
| GM12875 | √ |  |  |  |  |  |
| GM12878 | √ | √ | √ | √ | √ | √ |
| GM12891 | √ |  |  | √ |  |  |
| GM12892 | √ |  |  | √ |  |  |
| GM15510 | √ |  |  |  |  |  |
| GM18505 | √ |  |  |  |  |  |
| GM18526 | √ |  |  |  |  |  |
| GM18951 | √ |  |  |  |  |  |
| GM19099 | √ |  |  |  |  |  |
| GM19193 | √ |  |  |  |  |  |
| GM19238 | √ |  |  |  |  |  |
| GM19239 | √ |  |  | √ |  |  |
| GM19240 | √ |  |  |  |  |  |
| GM23338 |  |  |  |  |  | √ |
| H1-hESC | √ | √ | √ | √ | √ | √ |
| H9 |  |  |  |  |  | √ |
| HAc | √ |  |  |  |  |  |
| HAEpiC |  |  |  | √ |  |  |
| HA-sp | √ |  |  |  |  |  |
| HBMEC | √ |  |  |  |  |  |
| HCF |  |  |  | √ |  |  |
| HCFaa | √ |  |  |  |  |  |
| HCM | √ |  |  | √ |  |  |
| HCPEpiC | √ |  |  |  |  |  |
| HCPEpiC |  |  |  | √ |  |  |
| HCT-116 | √ |  |  | √ |  | √ |
| HEEpiC | √ |  |  | √ |  |  |
| HEK293 | √ |  |  | √ |  | √ |
| HEK293-T-REx | √ |  |  |  |  |  |
| HeLa-S3 | √ | √ |  | √ | √ | √ |
| Hepatocytes |  |  |  | √ |  |  |
| HepG2 | √ | √ | √ | √ | √ | √ |
| HFF | √ |  |  |  |  |  |
| HFF-Myc | √ |  |  |  |  |  |
| HIPEpiC |  |  |  | √ |  |  |
| HL-60 | √ |  |  | √ |  |  |
| HMEC | √ |  |  | √ |  |  |
| HMF | √ |  |  |  |  |  |
| HNPCEpiC |  |  |  | √ |  |  |
| HPAEpiC |  |  |  | √ |  |  |
| HPAF | √ |  |  |  |  |  |
| HPF | √ |  |  |  |  |  |
| HRCEpiC |  |  |  | √ |  |  |
| HRE | √ |  |  | √ |  |  |
| HRPEpiC | √ |  |  | √ |  |  |
| HSMM | √ |  |  |  |  |  |
| HSMMtube | √ |  |  |  |  |  |
| HUVEC | √ | √ |  | √ |  | √ |
| HVMF | √ |  |  |  |  |  |
| IMR90 | √ |  | √ | √ | √ | √ |
| Jurkat |  |  |  | √ |  |  |
| K562 | √ | √ | √ | √ | √ | √ |
| Karpas-422 |  |  | √ |  |  | √ |
| KMS-11 |  |  |  |  |  | √ |
| KOPT-K1 |  |  |  |  |  | √ |
| LNCaP |  |  |  | √ |  |  |
| Loucy |  |  |  |  |  | √ |
| MCF10A-Er-Src | √ |  |  | √ |  |  |
| MCF-7 | √ |  | √ | √ |  | √ |
| MM.1S |  |  |  |  |  | √ |
| NB4 | √ |  |  | √ |  |  |
| NH-A | √ |  |  | √ |  |  |
| NHBE |  |  |  | √ |  |  |
| NHDF-Ad | √ |  |  |  |  |  |
| NHDF-neo | √ |  |  | √ |  |  |
| NHEK | √ | √ |  |  |  |  |
| NHLF | √ | √ |  |  |  |  |
| NT2-D1 | √ |  |  | √ |  |  |
| OCI-LY1 |  |  |  |  |  | √ |
| OCI-LY3 |  |  |  |  |  | √ |
| OCI-LY7 |  |  | √ |  |  | √ |
| Osteobl | √ |  |  |  |  |  |
| ovcar-3 |  |  |  | √ |  |  |
| PANC-1 | √ |  |  | √ |  | √ |
| PBDE | √ |  |  |  |  |  |
| PBDEFetal | √ |  |  |  |  |  |
| PC-3 |  |  | √ |  |  | √ |
| PC-9 |  |  |  |  |  | √ |
| PFSK-1 | √ |  |  |  |  |  |
| PFSK-1 |  |  |  | √ |  |  |
| PrEC |  |  |  | √ |  |  |
| ProgFib | √ |  |  | √ |  |  |
| Raji | √ |  |  |  |  |  |
| RPTEC | √ |  |  | √ |  |  |
| RWPE1 |  |  |  |  |  | √ |
| RWPE2 | √ |  |  |  |  | √ |
| SAEC | √ |  |  | √ |  |  |
| SH-SY5Y |  |  |  |  |  |  |
| SKMC |  |  | √ | √ |  |  |
| SK-N-MC | √ |  |  | √ |  | √ |
| SK-N-SH | √ | √ |  | √ | √ | √ |
| SK-N-SH_RA | √ |  |  | √ |  |  |
| SUDHL6 |  |  |  |  |  | √ |
| T-47D | √ |  |  | √ |  |  |
| U2OS | √ |  |  |  |  |  |
| U87 | √ |  |  | √ |  |  |
| VCaP |  |  |  |  |  | √ |
| WERI-Rb-1 | √ |  |  |  |  |  |
| WI-38 | √ |  |  |  |  |  |
| A172 |  |  | √ |  |  |  |
| A375 |  |  | √ |  |  |  |
| Caki2 |  |  | √ |  |  |  |
| Daoy |  |  | √ |  |  |  |
| G401 |  |  | √ |  |  |  |
| H4 |  |  | √ |  |  |  |
| HT1080 |  |  | √ |  |  |  |
| HT-29 |  |  | √ |  |  |  |
| LHCN-M2 |  |  | √ |  |  |  |
| M059J |  |  | √ |  |  |  |
| MG63 |  |  | √ |  |  |  |
| NCI-H460 |  |  | √ |  |  |  |
| RPMI-7951 |  |  | √ |  |  |  |
| SJCRH30 |  |  | √ |  |  |  |
| SJSA1 |  |  | √ |  |  |  |
| SK-MEL-5 |  |  | √ |  |  |  |
| SK-N-DZ |  |  | √ |  |  |  |

**Supplementary Table 2. The siRNA sequences of FOXA1.**

| **Species** | **Primer name** | **Sequences** |
| --- | --- | --- |
| Homo sapiens | siFOXA1-186 | Forward: GCGACUGGAACAGCUACUATT  Reverse: UAGUAGCUGUUCCAGUCGCTT |
| Homo sapiens | siFOXA1-1415 | Forward: CGUACUACCAAGGUGUGUTT  Reverse: ACACACCUUGGUAGUACGCTT |
| Homo sapiens | siFOXA1-1520 | Forward: GCACUGCAAUACUCGCCUUTT  Reverse: AAGGCGAGUAUUGCAGUGCTT |
| Homo sapiens | Negative Control | Forward: UUCUCCGAACGUGUCACGUTT  Reverse: ACGUGACACGUUCGGAGAATT |

**Supplementary Table 3. Primers used in this study.**

| **Primer name** | **Sequences** |
| --- | --- |
| FOXA1 | Forward: ACCAGCCACCACATCATC  Reverse: TCAGAAACAAAGAGACAGAAGG |
| h_circ_0005188 | Forward: TATTTCAGCAATGCCCCTGT  Reverse: GCCCCACTGTCAGGAATACA |
| h_circ_0005188(2) | Forward: TATTTCAGCAATGCCCCTGT  Reverse: AGCCCCACTGTCAGGAATAC |
| h_circ_0003006 | Forward: TGCAGAGACAACAAGGATGG  Reverse: GTTTGGGATAGGCACGTCAT |
| h_circ_0008301 | Forward: TCCAGCTGCCATGGTGAT  Reverse: CGTGATGCGGAGGAAGTC |
| h_circ_0007769 | Forward: GAAGAGCCGACGAACAGAAC  Reverse: CTTTGAAGCTTTGGGACGAG |
| h_circ_0004565 | Forward: CCAACGACATTGAGGTACTAAAGA  Reverse: GAGCATCCCTATGGAGAGCA |
| h_circ_0006913 | Forward: ACCGGAAAACCCAAGAAACT  Reverse: CTGGAAATCCCTGATGTGCT |
| h_circ_0004004 | Forward: CAATCTGGTTCCGCTACGAC  Reverse: GTAGGAGTACCGCTGCTTGC |
| h_circ_0000515 | Forward: GTCAGACTGGGCAGGAGATG  Reverse: GACATGGGAGTGGAGTGACA |
| h_circ_0000515(2) | Forward: GTCAGACTGGGCAGGAGATG  Reverse: GGACATGGGAGTGGAGTGAC |
| h_circ_0000515(3) | Forward: TCAGACTGGGCAGGAGATG  Reverse: GACATGGGAGTGGAGTGACA |
| h_circ_0007099 | Forward: TCTCACGCTTTCTGCTCAAG  Reverse: TCTTCCCACTGCAAATACGA |
| h_circ_0007361 | Forward: CTCAGACCCAGGAGCAGAAT  Reverse: GATACCAGCCACTCCACACA |
| h_circ_0040777 | Forward: GGCCTCATTACCTTCTCACG  Reverse: TTTCGCTCCACATAAAATGC |
| h_circHIPK3 | Forward: TATGTTGGTGGATCCTGTTCGGCA  Reverse: TGGTGGGTAGACCAAGACTTGTGA |
| h_circMTO1 | Forward: GAGCTGTAGAAGATCTTATTC  Reverse: CACAGGCCATCCAAGGCATC |
| h_circMTO1-2 | Forward: GCCTGAACACACTGGGAAAT  Reverse: CACAGATGCGAGAACACAGG |
| GAPDH | Forward: GTCAGTGGTGGACCTGACCT  Reverse: AGGGGTCTACATGGCAACTG |
